# Mechanism of EC‐EXOs‐Derived THBS3 Targeting CD47 to Regulate BMSCs Differentiation to Ameliorate Bone Loss

**DOI:** 10.1111/cpr.70066

**Published:** 2025-06-13

**Authors:** Jiaojiao Wang, Zhaokai Zhou, Wenjie Chen, Yun Chen, Qiyue Zheng, Yajun Chen, Zhengxiao Ouyang, Ran Xu, Qiong Lu

**Affiliations:** ^1^ Department of Pharmacy The Second Xiangya Hospital, Central South University Changsha Hunan People's Republic of China; ^2^ National Clinical Research Center for Metabolic Diseases The Second Xiangya Hospital of Central South University Changsha Hunan China; ^3^ Institute of Clinical Pharmacy Central South University Changsha Hunan People's Republic of China; ^4^ Department of Urology The Second Xiangya Hospital of Central South University Changsha Hunan People's Republic of China; ^5^ School of Pharmaceutical Sciences Guangzhou University of Chinese Medicine Guangzhou People's Republic of China; ^6^ Department of Orthopedics The Second Xiangya Hospital of Central South University Changsha People's Republic of China

**Keywords:** ageing, bone marrow mesenchymal stem cells, CD47 antibody, osteogenic differentiation, osteoporosis, thrombospondin‐3, vascular endothelial cell exosomes

## Abstract

With the continuous increase of the elderly population and the deepening of population ageing in China, osteoporosis has gradually become one of the significant public health problems. Elucidating the pathophysiological mechanisms that induce osteoporosis and identifying more effective therapeutic targets is of great clinical significance. In this study, in vitro experiments demonstrated that endothelial cell exosomes (EC‐EXOs) promoted osteogenic and inhibited adipogenic differentiation of bone marrow mesenchymal stem cells (BMSCs). Aged and ovariectomy (OVX)‐induced osteoporosis mice models injected with EC‐EXOs confirmed that EC‐EXOs delayed bone loss. Proteomic analysis revealed a key protein regulating the differentiation of BMSCs. Expression of THBS3 was significantly higher in EC‐EXOs than in Human microvascular endothelial cells (HMEC‐1). In vitro and in vivo experiments further validated that THBS3 promoted BMSCs' osteogenic differentiation, inhibited their adipogenic differentiation, and retarded bone loss. Computational biology analysis found that CD47 is a downstream target and potentially functional receptor in BMSCs that bind to THBS3. THBS3 treatment of BMSCs down‐regulated the expression of CD47 in in vitro experiments. The aged/OVX models further confirmed that EC‐EXOs can regulate the differentiation of BMSCs and delay the process of bone loss via the THBS3–CD47 axis. CD47 antibody may be a potential therapeutic agent for treating ageing‐associated bone loss.

## Graphical abstract

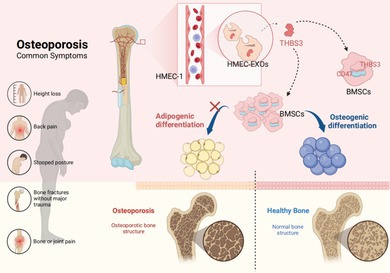

**Abbreviations**
BMSCs: bone marrow mesenchymal stem cells
EC‐EXOs: endothelial cell exosomes
GO: gene ontology
HE: Haematoxylin–eosin staining
HMEC‐1: human microvascular endothelial cells
IGF‐1: insulin‐like growth factor
KEGG: Kyoto Encyclopedia of Genes and Genomes
NTA: nanoparticle tracking analysis
OCN: osteocalcin
OVX: ovariectomy
PBS: phosphate buffered saline
PPI: protein–protein interaction networks
ROI: region of interest
RT‐qPCR: reverse transcription and quantitative real‐time polymerase chain reaction
TEM: transmission electron microscope
THBS3: thrombospondin‐3
TRAP: tartrate resistant acid phosphatase staining
WB: western blot

## 1 Introduction

As the population ages, a growing number of people face the risk of developing osteoporosis, which affects more than 200 million people worldwide [1]. Ageing leads to significant alterations in the skeleton, and age‐related bone loss increases the risk of fracture in the elderly, characterised by decreased bone formation and increased bone marrow fat [2]. The current clinical drugs in the treatment of osteoporosis mainly work by inhibiting bone resorption, with some promoting osteogenesis, such as bisphosphonates [3] and parathyroid hormone analogs. Nevertheless, the use of this drug for more than 2 years will increase the risk of bone tumours [4]. Therefore, there is an urgent need to explore more effective and safer bone‐enhancing drugs [5]. Bone marrow mesenchymal stem cells (BMSCs) possess the capacity to differentiate into a variety of cells, including osteogenic differentiation, adipogenic differentiation, and chondrogenic differentiation [6]. With ageing, the differentiation of BMSCs into osteoblasts slows down and bone formation capacity decreases, while adipogenic differentiation is enhanced and adipocytes increase in the bone marrow, leading to a decrease in bone mass, the occurrence of osteoporosis, and an increased risk of fracture [7]. Thus, this study attempted to identify therapeutic targets for ageing‐associated osteoporosis by deeply deciphering the molecular mechanisms regulating the relationship between osteogenesis and lipogenesis in BMSCs [8].

Exosomes, a natural endogenous nanocarrier, could participate in the exchange of information among cells during bone reconstruction through receptor‐mediated endocytosis [9]. Compared with general nanocarriers, exosomes could combine the advantages of nanoparticle size with no cytotoxicity, low immunogenicity, high target specificity, and high drug loading capacity, which opens up a promising avenue for the transport of genes and anti‐osteoporosis drugs [10]. It has been indicated that radiation‐induced BMSC‐derived exosomes restore the function of bone marrow mesenchymal stem cell receptors [11]. Qi et al. showed that mesenchymal stem cell‐derived exosomes could effectively repair bone defects via enhanced angiogenesis and osteogenesis [12]. There is a close spatial and temporal connection between bone and angiogenesis, namely, ‘angiogenesis‐osteogenesis coupling’ [13]. Yang et al. demonstrated that engineered exosomes enriched with Bmp2 mRNA, when delivered via hydrogels, significantly enhance bone regeneration [14], underscoring the therapeutic potential of tailored exosomal cargos. Parallel studies by Jin et al. revealed that BMSC‐derived exosomes mitigate osteoarthritis by suppressing chondrocyte senescence [15], highlighting their multifunctional roles in skeletal disorders. Human microvascular endothelial cells (HMEC‐1) can express various active molecules to regulate bone development and remodelling. Endothelial cell exosomes (EC‐EXOs) have been demonstrated to be more effective for bone targeting than osteoblast‐derived exosomes or bone marrow mesenchymal stem cell‐derived exosomes and could exert an inhibitory effect on osteoporosis by inhibiting osteoclast activity [16].

Proteins, metabolites, and nucleic acids delivered by exosomes to recipient cells can effectively alter the biological responses of the recipient cells, with the ability to regulate inter‐cellular communication and complex intra‐cellular pathways, thereby accelerating or inhibiting disease progression [17, 18]. Among them, exosomal proteins are involved in almost every step of exosome production, including endocytosis, exosome release, and interactions with receptor cells [17]. EC‐EXOs play a crucial role in bone remodelling; however, the mechanism by which EC‐EXOs influence bone remodelling through the regulation of osteogenic and adipogenic differentiation in BMSCs remains underexplored. This study aims to investigate the role of EC‐EXOs in osteoporosis, identify their downstream targets, and provide novel therapeutic insights and a theoretical basis for clinical osteoporosis treatment.

## 2 Materials and Methods

### 2.1 Extraction and Identification of EC‐EXOs

#### 2.1.1 Extraction of Primary BMSCs

The main reagents of this study are in Table S1. Four‐week‐old C57BL/6J mice (SCXK Xiang 2016‐0002) were decapitated and executed, and the femur and tibia on both sides of the mice were quickly removed to remove the bone surface tissues. The obtained bones were placed in α‐MEM (CellMax, China) basal medium to keep the bones moist so as not to affect the cell activity. Surgical scissors were used to remove the epiphyses at both ends of the bone, and the bone marrow cavity was repeatedly flushed with α‐MEM basal medium until the bone marrow cavity was translucent. The flushed cell suspension was collected, centrifuged at 1000 r/min for 5 min, resuspended using α‐MEM complete medium containing 15% fetal bovine serum (CellMax, China), transferred to a cell culture dish, and cultured in a 37°C, 5% CO_2_ cell culture incubator. After overnight incubation, the BMSCs were adherent to the wall, and cell exchange could be performed. Cell exchange was performed every 24 h for the first 3 days, and after 3 days, the cells could be exchanged at intervals, and when the cell confluence reached 80%–90%, cell passaging could be performed for subsequent experiments.

#### 2.1.2 Separation of EC‐EXOs

Take 240 mL of HMEC‐1 supernatant and add it to a 50 mL centrifuge tube, add phosphate‐buffered saline (PBS, Procell, China) to level it to 40 mL, centrifuge it at 4°C at 300× *g* at rotational speed for 10 min, and transfer the supernatant to a new centrifuge tube. The above supernatant was centrifuged at 4°C at 2000 g for 10 min and transferred to a 40 mL ultracentrifuge tube. After levelling, the mid‐supernatant was centrifuged at 10,000× *g* at 4°C for 30 min and transferred to a 40 mL ultracentrifuge tube. After levelling again, the middle supernatant was centrifuged at 100,000× *g* at 4°C for 70 min, the transparent precipitate was taken, and the precipitate was resuspended in 2400 μL of PBS, and the result was the exosome solution. Dispense the exosome solution and use it immediately or store it in the refrigerator at −80°C.

#### 2.1.3 Transmission Electron Microscope

The extracted EC‐EXOs solution was centrifuged, and 15 μl of EC‐EXOs suspension was adsorbed onto a copper grid and left for 1 min. EC‐EXOs on the copper mesh were blotted dry using filter paper and stained with 2% hydrogen peroxide acetate staining solution for 1 min at room temperature. EC‐EXOs on the copper mesh were again blotted with filter paper and baked under a lamp for 10 min. The samples were observed by transmission electron microscope (TEM).

#### 2.1.4 Nanoparticle Tracking Analysis (NTA)

The sample cell was washed with ultrapure water to prevent residual particles from affecting the results; the instrument was calibrated with polystyrene microspheres (100 nm); the sample cell was washed with 1× PBS buffer (SH30028.02, HyClone); the EC‐EXOs were diluted with 1× PBS buffer and injected for detection.

#### 2.1.5 Western Blot (WB)

Standards and diluted EC‐EXOs samples to be tested were taken for WB experiments (see Supporting Information).

#### 2.1.6 EC‐EXOs Tracing Experiment

The EC‐EXOs were removed from the refrigerator and placed on ice, melted, and then Diluent C was added to a total volume of 500 μL based on the volume of EC‐EXOs, mixed thoroughly, and labelled as tube A. The EC‐EXOs were then placed in a 1.5 mL sterile EP tube, and 500 μL of Diluent C and 4 μL of PKH67 (Sigma‐Aldrich, USA) were added. Meanwhile, 500 μL of Diluent C and 4 μL of PKH67 were added to a 1.5 mL sterile EP tube, mixed thoroughly, and labelled as tube B. The mixture of tubes A and B was then mixed to a total volume of 500 μL. Mix the above mixture of tubes A and B, mix thoroughly, and incubate at room temperature for 5 min. Add 1% BSA (Solarbio, China) to terminate the staining for 1 min, add 1% BSA until the volume of the mixture is 20 mL, and centrifuge at 120,000× *g* for 60 min in an ultra‐high‐speed centrifuge. The supernatant was discarded, and the PKH67‐labelled EC‐EXOs precipitate was dissolved in sterile PBS.

Discard the BMSCs culture medium in the biosafety cabinet, wash the BMSCs with sterile PBS, add trypsin (Procell, China) 1 mL for cell digestion, add fresh α‐MEM culture medium to terminate the digestion after digestion is completed, transfer the cell suspension into a centrifuge tube, centrifuge at 1000 r/min for 5 min, discard the supernatant, add fresh α‐MEM culture medium and blow into the cell suspension for cell counting. Put the sterile slide into a 24‐well plate, BMSCs were inoculated into the 24‐well plate at the rate of 4 × 104 per well, mixed well, and incubated overnight.

Discard the cell culture supernatant, add α‐MEM medium without exosome serum, add PKH67‐labelled EC‐EXOs to the wells, about 10 μg or so per well, mix well and place in the cell culture incubator for 48 h of total incubation. At the end of incubation, the medium was discarded, PBS was moistened and washed three times, BMSCs were fixed with 4% paraformaldehyde, paraformaldehyde was discarded, sterile PBS was moistened and washed 3 times, and BMSCs were treated with 0.1% Triton X‐100 in PBS for 5 min. Climbing tablets were washed with PBS three times, sealed with DAPI‐containing sealer, and photographed under laser confocal microscope observation.

### 2.2 Effect of EC‐EXOs on Osteogenic‐Adipogenic Differentiation of BMSCs

#### 2.2.1 Osteogenic‐Induced Differentiation of BMSCs

The BMSCs were inoculated into gelatin‐coated six‐well plates and cultured at 37°C with 5% CO_2_. BMSCs were seeded at a density of 3 × 10^5^ cells per well in 6‐well plates. When the fusion degree of BMSCs in the six‐well plates reached 60%–70% and 100%, respectively, under the condition of avoiding light, the normal α‐MEM complete medium was replaced with the prepared complete medium for osteogenic and lipogenic induction (see Supporting Information), and different cell experimental subgroups were treated (control/PBS group, 25 μg/mL EC‐EXOs group, 50 μg/mL EC‐EXOs group, 100 μg/mL EC‐EXOs group) to induce the differentiation of BMSCs to osteoblasts and adipocytes. The osteogenic and adipogenic induction medium (Sigma‐Aldrich, USA) was changed every 2–3 days, and the cells were cultured at 37°C with 5% CO_2_ for about 14–21 days. The time of termination of cell induction was decided according to the precipitation of calcium crystals and formation of mineralised nodules, and the number and size of lipid droplets formed by BMSCs induced by adipogenesis, respectively, and then stained for observation and quantification.

#### 2.2.2 Alizarin Red Staining and Quantification

Discard the cell culture medium in the six‐well plate, add PBS, wash gently for 1–2 times, add 2 mL of 4% paraformaldehyde (Biosharp, China) to each well, and fix for 30 min at room temperature. Discard paraformaldehyde, wash with PBS for 2–3 times, add 1 mL alizarin red staining solution (HaiXing, China) to each well, and leave to stain for 3–5 min. Discard the alizarin red staining solution, wash the staining solution with PBS several times until no excess staining solution remains, observe the formation of mineralized nodules under the microscope, and take photos. Preparation of 10% CPC solution: add 10% CPC (Sigma‐Aldrich, USA), Na_2_HPO_4_ (Sigma‐Aldrich, USA) with a final concentration of 10 mM in an appropriate amount of ultrapure water, dissolve it by ultrasonication, and add hydrochloric acid to adjust the pH value to 7.0. The liquid in the six‐well plate was discarded and dried as much as possible, 1 mL of 10% CPC solution was added, and the calcium salts and calcium nodules were eluted by gently shaking on a shaker for 15 min–1 h. Transfer the eluted CPC into an EP tube, dilute it 10 times, and add it to a 96‐well plate; zero with the same volume of 10% CPC. The OD value at 562 nm was determined.

#### 2.2.3 Oil Red O Staining and Quantification

Discard the cell culture medium in the six‐well plate, add PBS, wash gently for 1–2 times, add 2 mL of 4% paraformaldehyde (Biosharp, China) to each well, and fix for 30 min at room temperature. Configure the oil red O staining solution (HaiXing, China) according to the ratio of oil red storage solution: distilled water = 3:2, mix well, and filter. Discard paraformaldehyde, wash with PBS for 2–3 times, add 1 mL of oil red O staining solution to each well, and leave to stain for 30 min. Discard the oil red O staining solution, wash with PBS for 2–3 times until no excess staining solution remains, observe the formation of lipid droplets under the microscope, and take photos. Dispose of the liquid in the six‐well plate and dry it as much as possible, add 1 mL of isopropanol (Jinyueguan, China) cover the six‐well plate to prevent the volatilisation of the isopropanol, and shake the plate gently for 10–15 min to elute the lipid droplets. Transfer the eluted isopropanol into an EP tube, dilute it 10 times, and add it to the 96‐well plate; zero with the same volume of isopropanol. The OD value at 510 nm was determined.

#### 2.2.4 Reverse Transcription and Quantitative Real‐Time Polymerase Chain Reaction

BMSCs were treated with 50 μg/mL EC‐EXOs, and osteogenesis was induced for 7 days in the osteogenic induction medium. qPCR was performed to detect the expression levels of genes related to osteogenic differentiation of BMSCs (see Tables S2–S5), and to assess the effect of EC‐EXOs on the osteogenic differentiation of BMSCs.

#### 2.2.5 Animal Experimental Grouping and Specimen Collection

Aged mouse model (average weight about 28 g): 15‐month‐old male C57BL/6J mice were divided into 2 groups of 8 mice each; the control group was injected with PBS (200 μL) in the tail vein, and the experimental group was injected with EC‐EXOs (150 μL/200 μL) in the tail vein for 5 consecutive weeks. Ovariectomy (OVX) mice osteoporosis model (average weight about 20 g) [19]: 8‐week‐old female C57BL/6J mice were divided into 3 groups of 8 mice each; the sham‐operated (SHAM) group was injected with PBS (200 μL) in the tail vein, the OVX control group was injected with PBS (200 μL) in the tail vein, and the OVX experimental group was injected with EC‐EXOs (150 μL/200 μL) in the tail vein, and the mice were injected for 5 consecutive weeks. Bone tissue collection: mice were decapitated and executed, both hind legs were removed, the skin and muscle tissue of the bones were removed, and the femur and tibia were placed in 4% paraformaldehyde (Biosharp, China) for fixation after being cleaned. Organ harvesting: mice were dissected, and the heart, liver, spleen, lungs, and kidneys were removed and rinsed quickly in saline, dried on filter paper, and fixed in 4% paraformaldehyde. Uterus removal was required for OVX modelling mice to confirm the success of OVX modelling.

#### 2.2.6 Mirco‐CT Analysis of Bone Histomorphometry

Soft tissues around the clean femur of mice were removed, then the femur was placed with the long axis parallel to the long axis of the scanning bed and the samples were scanned using a Micro‐CT scanner. All images were processed manually to separate cancellous and cortical bone and preserve their morphology. Scans were repeated three times and bone microstructural parameters were obtained from the same region of interest (ROI) for each group. Image reconstruction software, data analysis software (Bruker micro‐CT), and 3D model visualisation software (Bruker micro‐CT) were used to analyse the structural parameters of the ROI, including bone volume fraction (BV/TV), number of trabeculae (Tb.N), trabeculae separation (Tb.Sp) and trabeculae thickness (Tb.Th).

#### 2.2.7 HE Staining, TRAP Staining, and OCN Immunohistochemical Staining

The wax block was placed in a paraffin slicer, and the slices were spread on a spreader with warm water at 40°C. Afterwards, the tissues were fished out with slides and the slices were baked in a 60°C oven. Paraffin sections of bone tissue were prepared for Haematoxylin–eosin (HE) staining (Servicebio, China), tartrate resistant acid phosphatase (TRAP) staining (Servicebio, China), and Osteocalcin (OCN) immunohistochemical staining (Servicebio, China) (Tables S6 and S7).

### 2.3 Identification of Key Proteins and Downstream Targets of EC‐EXOs Targeting BMSCs

HMEC‐1 samples and culture supernatants were collected for bioinformatics analysis to screen for differential proteins between EC‐EXOs and HMEC‐1. The quantitative study of the proteins was based on a series of cutting‐edge technologies. The technical route is as follows: protein extraction → proteolysis → TMT labelling → high‐performance liquid chromatography separation → liquid tandem mass spectrometry analysis → database search → bioinformatic analysis. Differentially expressed proteins were analysed for EC‐EXOs and HMEC‐1, and the comparative groups were screened to obtain differentially expressed proteins according to the relative quantitative value of greater than 1.3‐fold or less than 1/1.3 and the statistical *T*‐test of *p* < 0.05, with significant up‐regulation when the relative quantitative value was > 1.3 and significant down‐regulation when the relative quantitative value was < 1/1.3. The overall distribution of differentially expressed proteins was further understood by drawing volcano plots.

Gene ontology (GO), which includes biological processes, cellular composition, and molecular functions, is an important biological function enrichment method and tool to elucidate the biological roles of proteins from different perspectives. Kyoto Encyclopedia of Genes and Genomes (KEGG) is a database resource that connects information networks of known molecular interactions such as metabolic pathways and biochemical reactions. We performed GO and KEGG enrichment analysis of differentially expressed proteins of EC‐EXOs and HMEC‐1 to explore the potential molecular mechanisms. Subsequently, the key proteins were further verified by quantitative real‐time polymerase chain reaction (RT‐qPCR).

### 2.4 Effect of Thrombospondin‐3 on Osteogenic‐Adipogenic Differentiation of BMSCs

The above studies suggest that Thrombospondin‐3 (THBS3) may play an important regulatory role in the development, growth, remodelling, and senescence of bone tissue. Whether THBS3 is involved in regulating the osteogenic–adipogenic differentiation of BMSCs in bone marrow has not been reported. Therefore, we further explored in depth the role of THBS3 on the osteogenic–adipogenic differentiation of BMSCs in in vitro cellular experiments and in in vivo aged and OVX mice.

We treated BMSCs cells with THBS3 (50 ng/mL), and PBS as experimental and control groups, respectively. Then, we carried out the induction of osteogenic and adipogenic differentiation of BMSCs, and performed alizarin red staining and quantification, oil red O staining and quantification, RNA extraction, and RT‐qPCR detection methods. 15‐month‐old male C57BL/6J mice were divided into two groups of 8 mice each; the control group was injected with PBS (200 μL) in the tail vein, and the experimental group was injected with THBS3 (50 μL/kg) in the tail vein for 5 weeks. OVX mouse osteoporosis model: 8‐week‐old female C57BL/6J mice were divided into three groups of 8 mice each; the sham‐operated group was injected with PBS (200 μL) in the tail vein, the OVX control group was injected with PBS (200 μL) in the tail vein, and the OVX experimental group was injected with THBS3 (50 μL/kg) in the tail vein, and the injection was carried out for 5 consecutive weeks. After the successful construction of the animal model, the specimens were collected for Micro‐CT analysis, HE staining, TRAP staining, and OCN immunohistochemical staining.

### 2.5 Identification of THBS3 Downstream Targets

BMSCs treated with THBS3 and PBS, respectively, were collected for transcriptome sequencing analysis, including data evaluation and quality control, RNAseq sequencing evaluation, gene structure analysis, expression level analysis, expression difference analysis, and enrichment analysis. We attempted to screen potential functional receptors in BMSCs that may bind to THBS3 by computational biology analysis.

KEGG pathway diagrams graphically illustrate metabolic pathways and the relationships between pathways. The labelling of differential genes on the corresponding pathways can visually reflect the impact of gene expression differences in metabolic pathways. Protein–protein interaction networks (PPI) are an analysis that describes the interactions between a group of proteins and can be used to screen out proteins that are located at key nodes of the PPI network, thereby guiding the screening of biomarkers. PPI was obtained using the STRING database (https://cn.string‐db.org) and further optimised using Cytoscape software. The GeneCards database (https://www.genecards.org/) was used to screen for osteoporosis disease‐related targets of action. Subsequently, GeneCards analyses, differential genes, and genes from PPIs were intersected to screen for key downstream targets of THBS3. The results were further verified by qPCR experiments. BMSCs were treated with THBS3 to investigate the expression of downstream targets.

### 2.6 Impact of the THBS3 Downstream Target CD47 on BMSCs

15‐month‐old male C57BL/6J mice were divided into three groups of 8 mice each; the control group was injected with PBS (200 μL) in the tail vein, the CD47 antibody group was injected with CD47 antibody (50 μg/200 μL) in the tail vein, and the THBS3 combined with CD4 antibody group was injected with THBS3 (50 μg/kg) + CD47 antibody (50 μg/200 μL), injected continuously for 5 weeks. OVX mouse osteoporosis model: 8‐week‐old female C57BL/6J mice were divided into four groups of 8 mice each; the sham surgery (SHAM) group was injected with PBS (200 μL) in the tail vein, the OVX control group was injected with PBS (200 μL) in the tail vein, the OVX CD47 antibody group was injected with CD47 antibody (50 μg/200 μL) in the tail vein, and the OVX THBS3 combined with CD47 antibody group was injected with THBS3 (50 μg/kg) + CD47 antibody in the tail vein (50 μg/200 μL) for 5 weeks. After successfully constructing animal experimental models, the specimens were collected for bone histomorphological Micro‐CT analysis, HE staining and TRAP staining experiments, and OCN immunohistochemical staining in turn.

### 2.7 Statistical Methods

Micro CT images were analysed for osteometric parameters using SkyScan (CTAn and DataViewer). GraphPad Prism 8 software was used for statistical analysis and graphing of data. The statistical analysis of qPCR data involved normalisation to the geometric mean of two validated reference genes (GAPDH and β‐actin) followed by relative quantification using the ΔΔCt method, with fold changes calculated as 2^−ΔΔCt2^; normality was assessed via Shapiro–Wilk testing (*p* > 0.05), and group comparisons were performed using unpaired Student's *t*‐test (two groups) or one‐way ANOVA with Bonferroni correction (multiple groups), with data presented as mean ± SEM from three independent biological replicates (*n* = 3). The optical density values of the target bands were analysed using Image J software. *p* < 0.05 was statistically significant (*p < 0.05, **p < 0.01, ***p < 0.001, #not statistically significant).

## 3 Results

### 3.1 Effect of EC‐EXOs on Osteogenic‐Lipogenic Fate Differentiation in BMSCs

**FIGURE 1:**
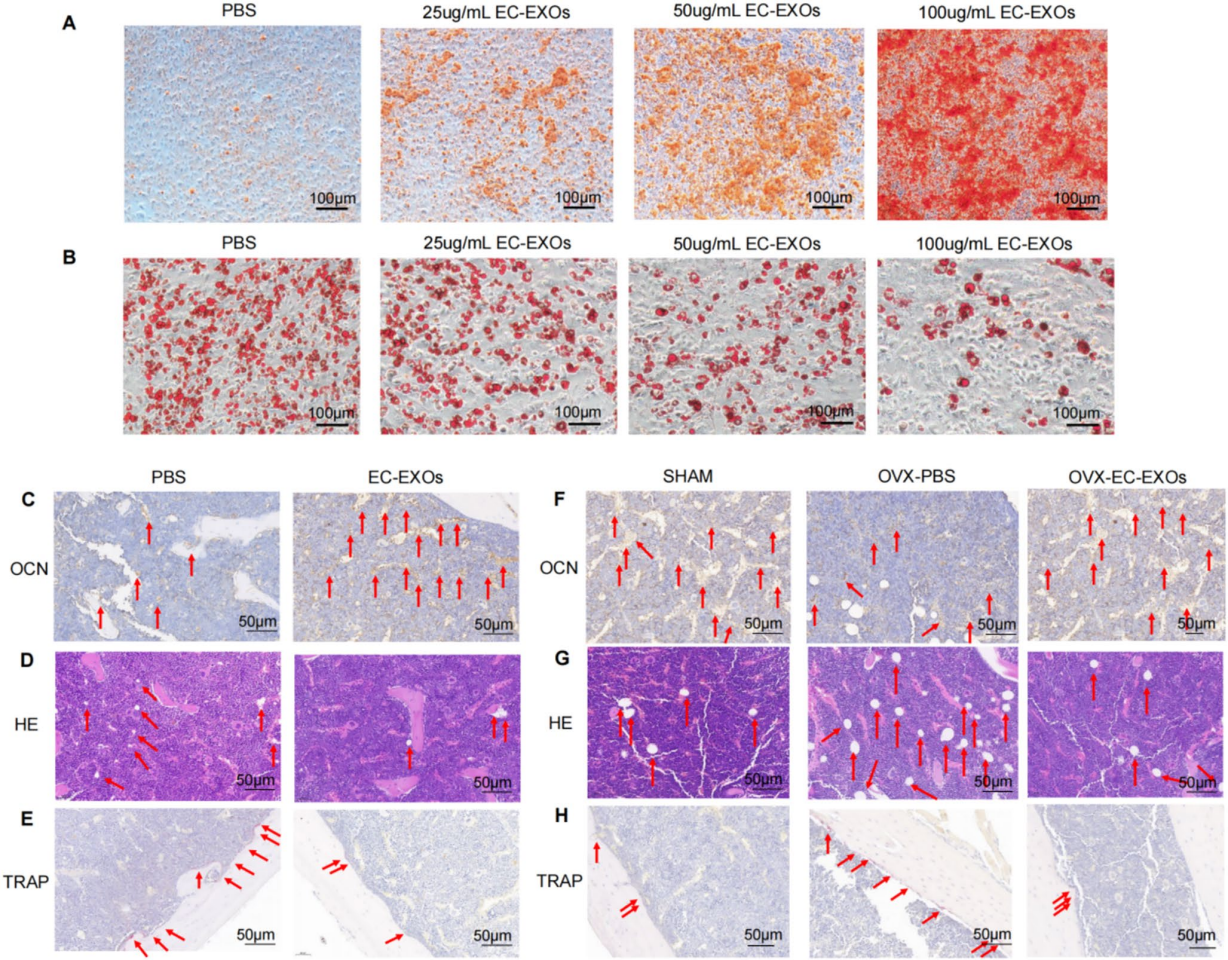
Effect of EC‐EXOs on osteogenic‐lipogenic fate differentiation in BMSCs. (A) Microscopic view of Alizarin‐red staining and (B) quantitative analysis demonstrating the effect of HMEC‐EXOs treatment on mineralised nodules and calcium salt formation in bone marrow mesenchymal stem cells (BMSCs); (C) Osteocalcin (OCN) staining shows osteoblasts deposited on the bone surface of aged mice in which the nuclei are blue and the cytoplasm is brown; (D) Haematoxylin–eosin (HE) staining shows fat vacuoles distributed in white regular circles in the bone marrow cavity of aged mice; (E) tartrate resistant acid phosphatase (TRAP) staining shows osteoclasts deposited on the bone surface of aged mice, and the nucleus of the cells is light blue and the cytoplasm is purplish red; (F) OCN staining, (G) HE staining and (H) TRAP staining in the SHAM and OVX groups.

As consistent with our previous findings [20], typical exosome‐like structures in EC‐EXOs would be taken up by BMSCs. EC‐EXOs exhibited characteristic cup‐shaped morphology with a mean diameter of 148.9 nm, expressing canonical exosomal markers CD63, CD9, and TSG101 while lacking endoplasmic reticulum contamination (calnexin‐negative). Functionally, PKH26‐labelled EC‐EXOs were efficiently internalised by BMSCs, confirming their bioactivity. Alizarin red staining revealed that mineralised nodules and calcium salt formation of BMSCs in the EC‐EXOs‐treated group was significantly higher than that in the PBS‐treated group. The degree of mineralised nodules and calcium salt formation increased with the increase of EC‐EXOs concentration (Figure 1A). Oil red O staining showed that the lipid droplet formation of BMSCs in the EC‐EXOs‐treated group was significantly lower than that in the PBS‐treated group. The lipid droplet formation situation decreased with the increase of EC‐EXOs concentration (Figure 1B). These results suggested that EC‐EXOs might promote the differentiation of BMSCs toward osteoblasts in a concentration‐dependent manner, and EC‐EXOs might inhibit the differentiation of BMSCs toward adipocytes in a concentration‐dependent manner.

In aged and OVX mice, OCN staining showed that the number of osteoblasts deposited on the surface of cancellous bone in the EC‐EXOs‐treated group was significantly greater than the number of osteoblasts deposited in the control group (Figure 1C,F), thus confirming that EC‐EXOs can promote bone formation by promoting osteoblast formation. Additionally, HE staining indicated that the number of fat vacuoles in the bone marrow cavity of the EC‐EXOs‐treated group was significantly smaller than that in the control group (Figure 1D,G). TRAP staining showed that the number of osteoclasts deposited on the surface of cancellous bone in the EC‐EXOs‐treated group was less than the control group (Figure 1E,H). These results confirmed that EC‐EXOs treatment reduced the number of adipocytes and osteoclasts in aged mice and delayed bone loss in OVX mice.

### 3.2 Analysis of EC‐EXOs and HMEC‐1 Differentially Expressed Proteins

**FIGURE 2:**
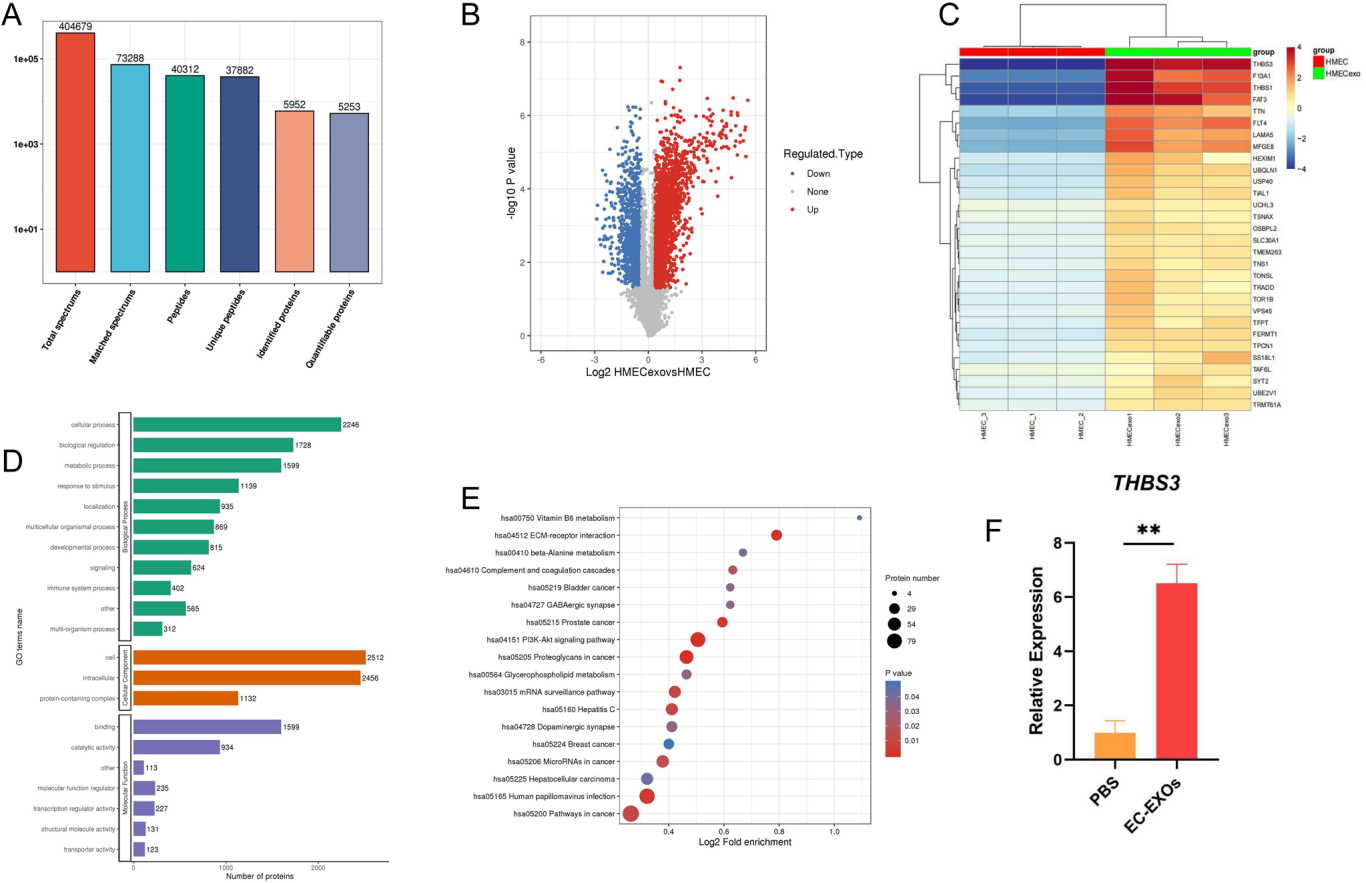
Identification of key proteins of EC‐EXOs affecting fate differentiation of BMSCs. (A) Quantitative identification of differentially expressed proteins of EC‐EXOs and HMEC‐1; (B) volcano plot of EC‐EXOs and HMEC‐1 differentially expressed proteins; (C) Heatmap of differentially expressed proteins of EC‐EXOs and HMEC‐1; (D, E) GO and KEGG analysis of differentially expressed proteins; (F) qPCR detected the effect of EC‐EXOs treatment on THBS3 expression in BMSCs. *p < 0.05, **p < 0.01, ***p < 0.00.

To further analyse in depth the possible mechanisms by which EC‐EXOs function, we analysed the differentially expressed proteins in EC‐EXOs and HMEC‐1 samples by bioinformatics. Mass spectrometry analysis yielded 404,679 secondary spectra, and a total of 40,312 peptides were identified by spectral resolution, with 37,882 specific peptides, and a total of 5952 proteins were identified, of which 5253 were quantifiable (Figure 2A). A total of 1725 significantly up‐regulated and 1042 significantly down‐regulated differential proteins were identified (Figure 2B). Compared with HMEC‐1, the expression of THBS3 was very high in EC‐EXOs, and THBS3 was selected for further validation in subsequent experiments (Figure 2C). Additionally, Figure 2D,E showed GO analysis and KEGG analysis of differentially expressed proteins. qPCR showed that the expression of THBS3 was significantly up‐regulated in BMSCs in the EC‐EXOs‐treated group compared with the control (PBS) group (Figure 2F). Therefore, EC‐EXOs regulating BMSCs differentiation may act through THBS3.

### 3.3 In Vitro Experiments to Verify the Effect of THBS3 on the Osteogenic‐Adipogenic Differentiation Capacity of BMSCs

**FIGURE 3:**
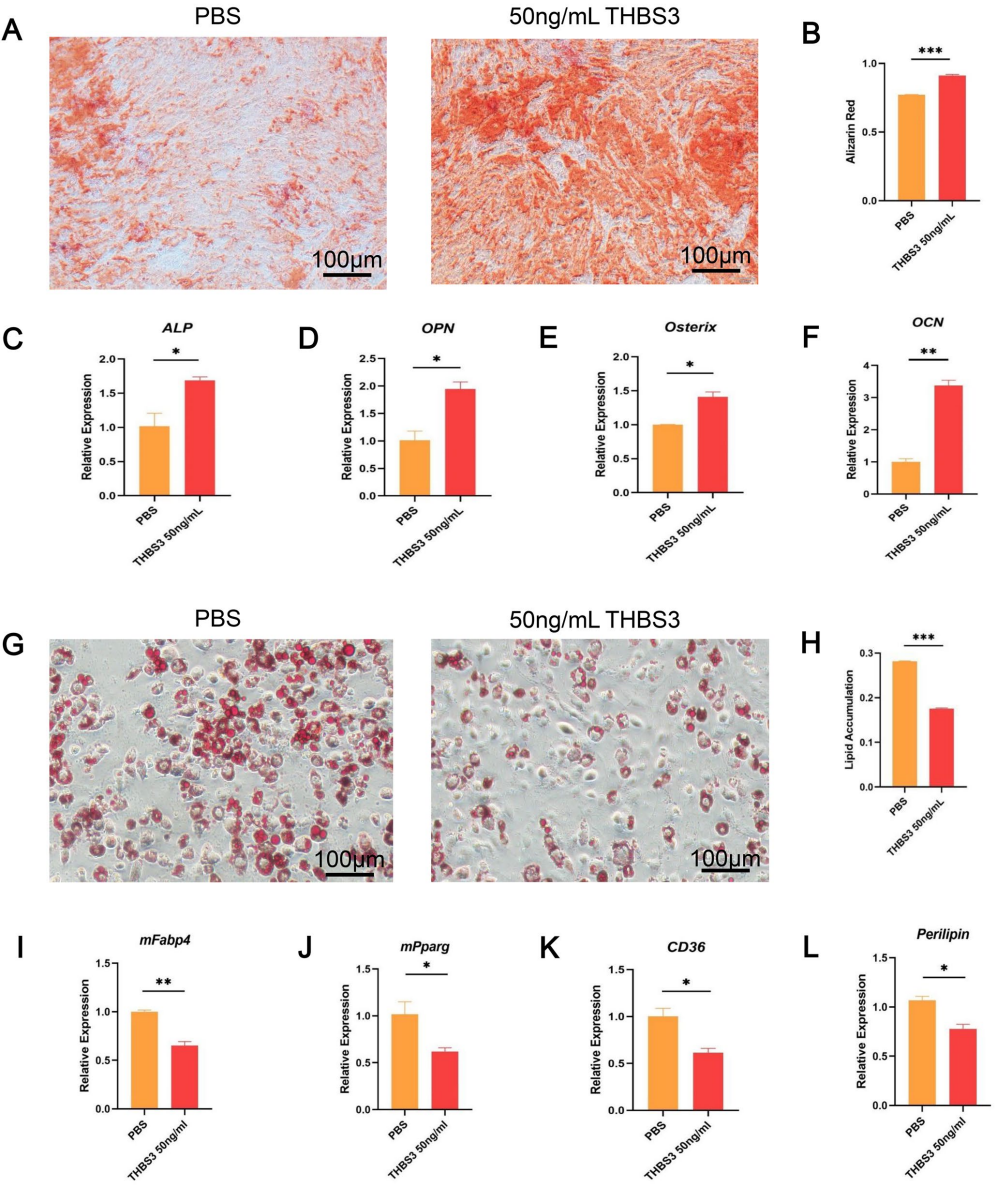
In vitro experiments to verify the effect of THBS3 on the osteogenic‐adipogenic differentiation capacity of BMSCs. (A) Alizarin‐red staining and (B) quantitative analysis indicated the effect of THBS3 treatment on mineralized nodules and calcium salt formation in BMSCs; (C–F) qPCR detected the effect of THBS3 treatment on the expression of genes related to osteogenic differentiation (ALP, OPN, Osterix and OCN) in BMSCs; (G) oil red O staining and (H) quantitative analysis indicated the effect of THBS3 treatment on lipid droplet formation in BMSCs; (I–L) qPCR detection of the effect of THBS3 treatment on the expression of genes related to lipid differentiation (mFabp4, mPparg, CD36, Perilipin) in BMSCs. Scale bar: 100 μm, *p < 0.05, **p < 0.01, ***p < 0.001.

In vitro and in vivo experiments were conducted to investigate whether the effects of EC‐EXOs on BMSC differentiation are mediated through THBS3. In vitro, BMSCs were treated with THBS3 under osteogenic and lipogenic induction conditions, respectively. Under osteogenesis‐inducing conditions, alizarin red staining and quantitative analysis revealed a significant increase in the formation of mineralized nodules and calcium deposits in THBS3‐treated BMSCs compared to control groups (Figure 3A,B). Furthermore, qPCR analysis confirmed that THBS3 treatment upregulated the expression of osteogenic differentiation markers, including ALP, OPN, Osterix, and OCN (Figure 3C–F). Under lipogenesis‐inducing conditions, THBS3 treatment markedly reduced lipid droplet formation in BMSCs (Figure 3G,H). Consistently, qPCR demonstrated downregulation of adipogenic differentiation‐related genes, such as mFabp4, mPparg, CD36, and Perilipin (Figure 3I–L). These findings indicated that THBS3 effectively promoted osteogenic differentiation while inhibiting adipogenic differentiation in BMSCs, supporting its critical role in regulating the differentiation of BMSCs.

### 3.4 In Vivo Experiments to Verify the Role of THBS3 in the Aged and OVX Mice

**FIGURE 4:**
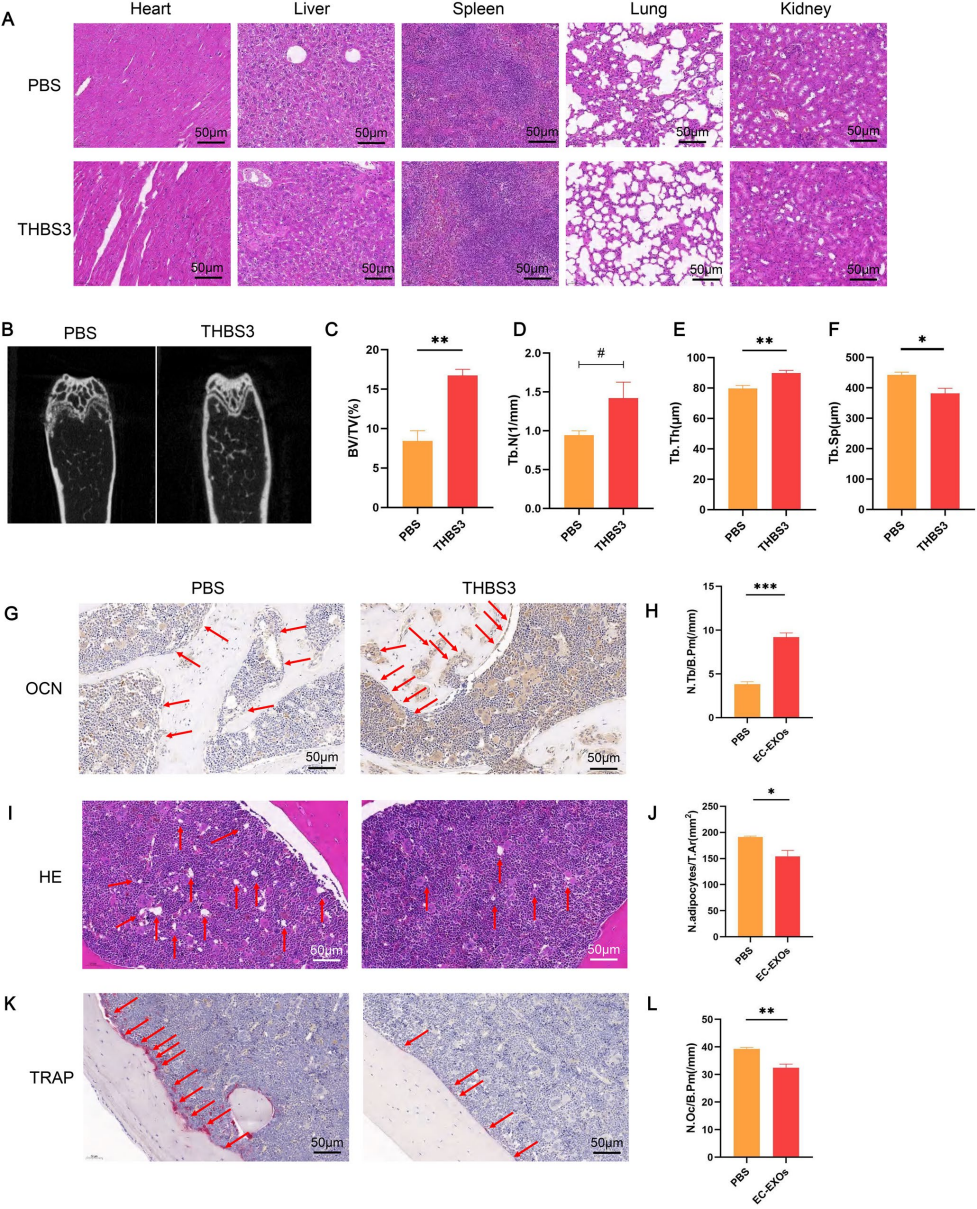
In vivo experiments to validate the role of THBS3 in the aged mice. (A) HE staining of pathological sections of heart, liver, spleen, lungs and kidneys; (B) Micro CT of the distal femur in aged mice; (C–F) Micro CT osteometric parameters (BV/TV: bone volume fraction, Tb. N: number of trabeculae, Tb. Th: trabeculae thickness, and Tb. Sp: trabeculae separation); (G) OCN staining showed osteoblasts deposited on the bone surface with blue nuclei and tan cytoplasm; (H) quantitative analysis of the ratio of the number of osteoblasts deposited on the bone surface to the area of the region; (I) HE staining showed the distribution of fat vacuoles in the bone marrow cavity as white regular circles; (J) quantitative analysis of the number of fat vacuoles in the bone marrow cavity versus regional area ratio; (K) TRAP staining indicated osteoclasts deposited on the bone surface, with light blue nuclei and burgundy cytoplasm; (L) quantitative analysis of the number of osteoclasts deposited on the bone surface versus the regional area ratio. *p < 0.05, **p < 0.01, ***p < 0.001.

In in vivo experiments, we utilised aged and OVX models to further elucidate the role of THBS3. Compared with the control group, the THBS3 group had normal physiological conditions in the heart, liver, spleen, lungs or kidneys, indicating excellent biocompatibility (Figure 4A). Micro‐CT imaging of femurs revealed significantly more cancellous bone in the THBS3 group than in the control group (Figure 4B). Additionally, Figure 4C–F showed that THBS3 treatment increased bone volume fraction and trabecular thickness and decreased trabecular separation in aged mice. OCN immunohistochemistry showed increased osteoblast deposition on the cancellous bone surface following THBS3 treatment, corroborating its role in promoting osteoblast formation and bone synthesis (Figure 4G,H). HE and TRAP staining further confirmed that THBS3 treatment reduced adipocyte accumulation and osteoclast numbers in the bone marrow of aged mice (Figure 4I–L).

**FIGURE 5:**
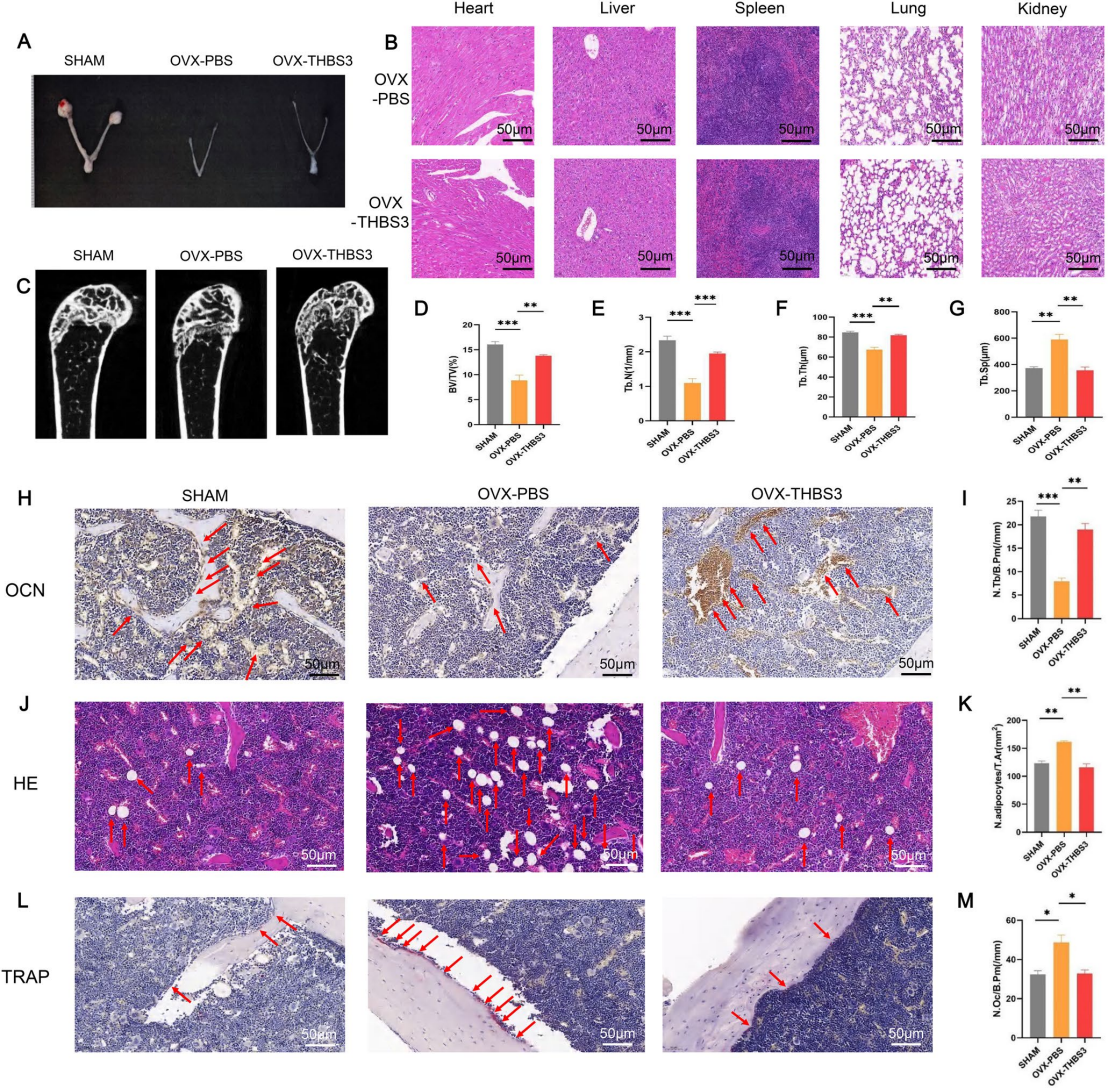
In vivo experiments to validate the effect of THBS3 on the process of osteoporosis in OVX mice. (A) The uterus of SHAM and OVX groups; (B) HE staining of pathological sections of heart, liver, spleen, lung and kidney; (C) Micro CT of the distal femur in SHAM and OVX groups; (D–G) Micro CT osteometric parameters in SHAM and OVX groups; (H) OCN staining indicated osteoblasts deposited on the bone surface; (I) quantitative analysis of the ratio of the number of osteoblasts deposited on the bone surface to the area; (J) HE staining showing the distribution of fat vacuoles in the bone marrow cavity; (K) quantitative analysis of the ratio of the number of fat vacuoles in the bone marrow cavity to the area of the area; (L) osteoclasts deposited on the bone surface by TRAP staining; (M) quantitative analysis of the ratio of the number of osteoclasts deposited on the bone surface to the area of the region. *p < 0.05, **p < 0.01, ***p < 0.001.

No obvious abnormality was seen in the uterus of the sham group, and the uterus of both the OVX‐PBS group and OVX‐THBS3 group was significantly atrophied, showing that the modelling of OVX experimental mice was successful (Figure 5A). Likewise, the THBS3 group had excellent biocompatibility (Figure 5B). THBS3 treatment increased bone volume fraction, number of trabeculae, and thickness of trabeculae and decreased trabecular separation in OVX mice (Figure 5C–G). THBS3 treatment increased the deposition of osteoblasts (Figure 5H,I), decreased the number of fat vacuoles in the bone marrow cavity (Figure 5J,K), and decreased the number of osteoclasts deposited on the surface of cancellous bone in OVX mice (Figure 5L,M). Collectively, these findings underscore the capacity of THBS3 to mitigate age‐related bone loss and delay the progression of osteoporosis in OVX mice by promoting osteoblast activity and reducing adipogenesis and osteoclastogenesis.

### 3.5 Computational Biology Analysis of Potential Downstream Targets of THBS3

**FIGURE 6:**
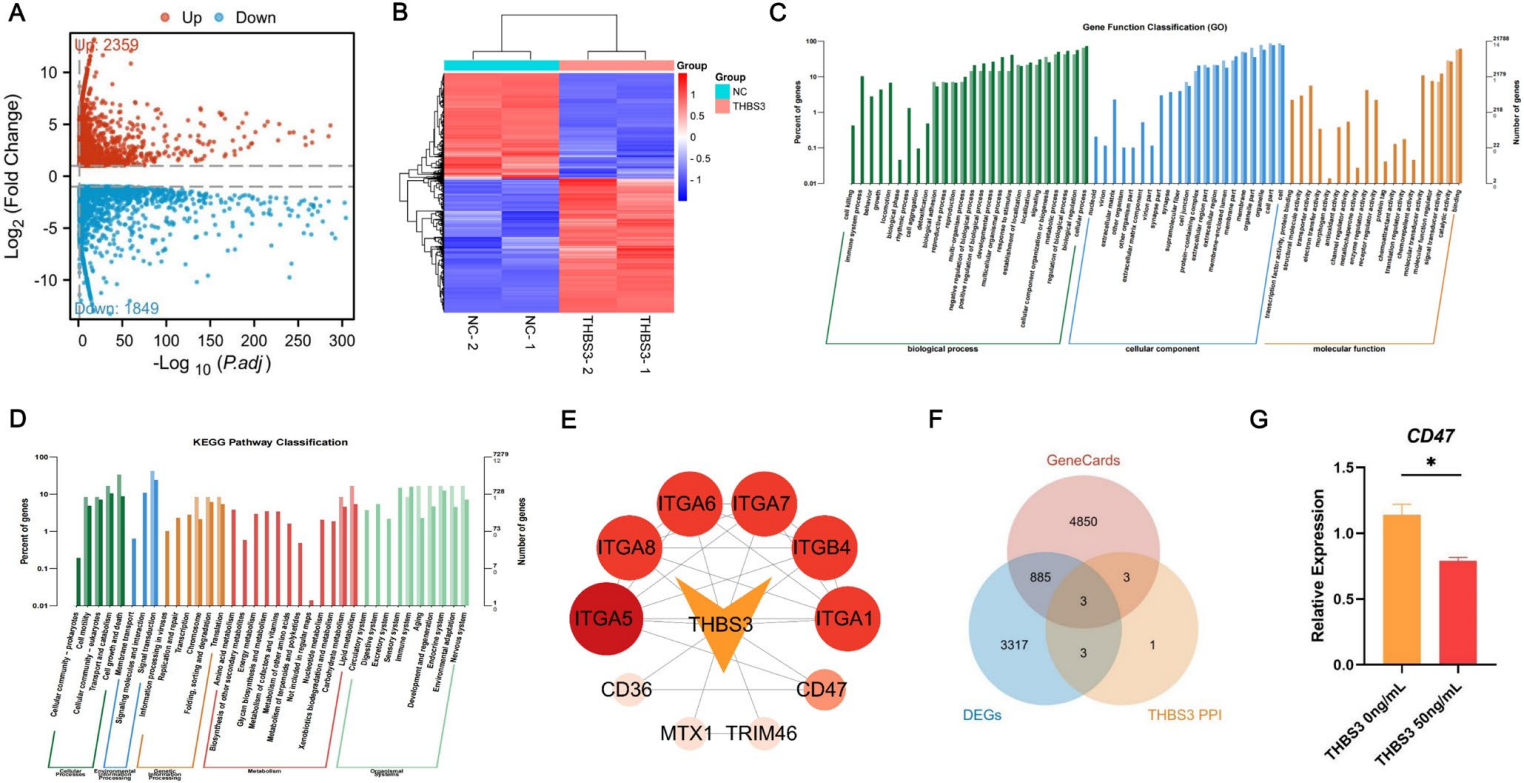
Detection of THBS3 downstream target CD47. (A, B) Volcano plot and Heatmap of differentially expressed genes in THBS3 versus PBS‐treated BMSCs; (C, D) GO and KEGG analysis of differentially expressed genes; (E) protein–protein interaction networks (PPI) of THBS3; (F) Venn plot of differential genes, PPIs, and osteoporosis gene targets; (G) qPCR detected the effect of THBS3 treatment on CD47 gene expression.

To further elucidate the mechanisms underlying the effects of THBS3 on the bone marrow microenvironment, transcriptome sequencing was conducted to compare THBS3‐treated BMSCs with PBS‐treated control BMSCs. Differential expression analysis identified 2359 upregulated and 1849 downregulated genes (Figure 6A,B). Functional enrichment analyses highlighted key biological processes and pathways influenced by THBS3 (Figure 6C,D). Additionally, PPI network analysis was performed to identify potential downstream targets of THBS3 (Figure 6E). GeneCards analysis identified 5741 osteoporosis‐related genes. A Venn diagram integrating these with the PPI network and differentially expressed genes highlighted three candidate targets: CD47, Itgb1 and Itga2b (Figure 6F). To validate these findings, qPCR analysis showed that CD47 expression was significantly downregulated in THBS3‐treated BMSCs compared to the control group (Figure 6G). These results suggest that THBS3 may regulate BMSC differentiation by targeting CD47, thereby playing a crucial role in modulating the bone marrow microenvironment.

### 3.6 In Vivo Validation of CD47 as a Downstream Target of THBS3 in the Aged and OVX Mice

**FIGURE 7:**
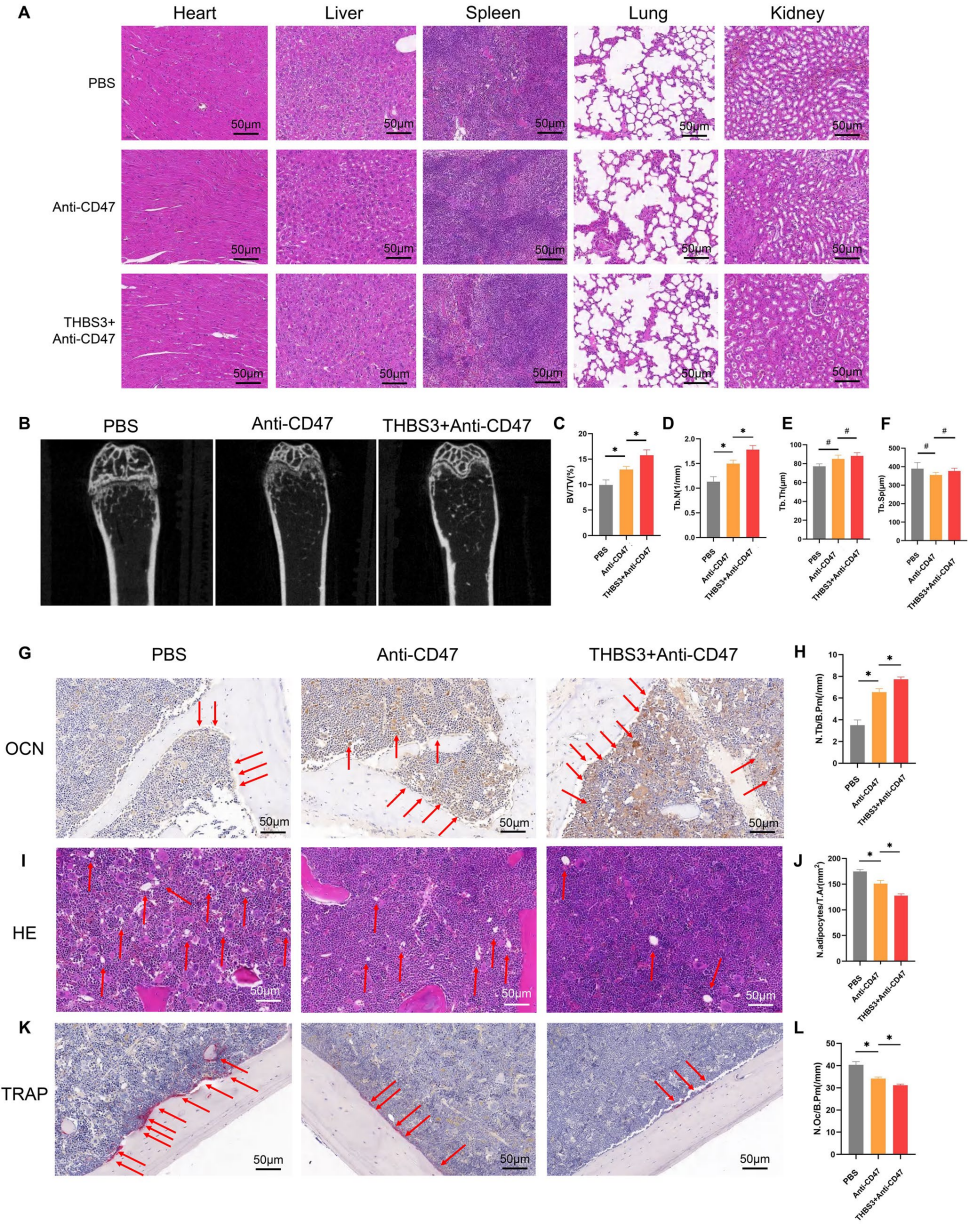
In vivo experiments to validate the role of THBS3 downstream target CD47 in the aged mice. (A) HE staining of pathological sections of heart, liver, spleen, lungs and kidneys; (B) Micro CT of the distal femur in aged mice; (C–F) Micro CT osteometric parameters; (G) OCN staining showing osteoblasts deposited on the bone surface; (H) quantitative analysis of the ratio of the number of osteoblasts deposited on the bone surface to the area of the region; (I) HE staining indicating the distribution of fat vacuoles in the bone marrow cavity; (J) quantitative analysis of the number of fat vacuoles in the bone marrow cavity versus regional area ratio; (K) TRAP staining indicating osteoclasts deposited on the bone surface; (L) quantitative analysis of the number of osteoclasts deposited on the bone surface versus the regional area ratio. *p < 0.05, **p < 0.01, ***p < 0.001.

To investigate whether the effects of THBS3 on the bone marrow microenvironment are mediated via CD47, we conducted in vivo experiments using aged and OVX mouse models. Mice models were administered tail vein injections of either CD47 antibody alone or the combination of THBS3 and CD47 antibody to evaluate the role of CD47. HE staining confirmed that both CD47 antibody and the combination treatment of CD47 antibody with THBS3 were non‐toxic and biocompatible across major organs, including the heart, liver, spleen, lungs, and kidneys in aged mice (Figure 7A). Micro‐CT showed that the number of cancellous bone in the Anti‐CD47 group was significantly more than that of the control group, and the number of cancellous bone in the THBS3 + Anti‐CD47 group was more than that in the Anti‐CD47 group (Figure 7B). Osteometric parameters revealed that CD47 treatment increased the bone volume fraction and the number of bone trabeculae; the THBS3 + Anti‐CD47 group increased the bone volume fraction and the number of bone trabeculae more significantly compared with the Anti‐CD47 group alone (Figure 7C–F). OCN staining showed that the number of osteoblasts deposited on the surface of cancellous bone in the Anti‐CD47 group was significantly more than the number of osteoblasts deposited in the control group, and the number of osteoblasts deposited on the surface of cancellous bone in the THBS3 + Anti‐CD47 group was more than the number of osteoblasts deposited in the Anti‐CD47‐alone group (Figure 7G,H). In the HE staining, the number of fat vacuoles in the bone marrow cavity in the Anti‐CD47 group was significantly smaller than that of the control group, and the number of fat vacuoles in the THBS3 + Anti‐CD47 group was significantly smaller than that in the Anti‐CD47 group (Figure 7I,J). TRAP staining showed that the number of osteoclasts deposited on the surface of cancellous bone in the Anti‐CD47 group was less than the number of osteoclasts deposited in the control group, and the number of osteoclasts deposited in the THBS3 + Anti‐CD47 group was less than that deposited in the Anti‐CD47 group (Figure 7K,L).

**FIGURE 8:**
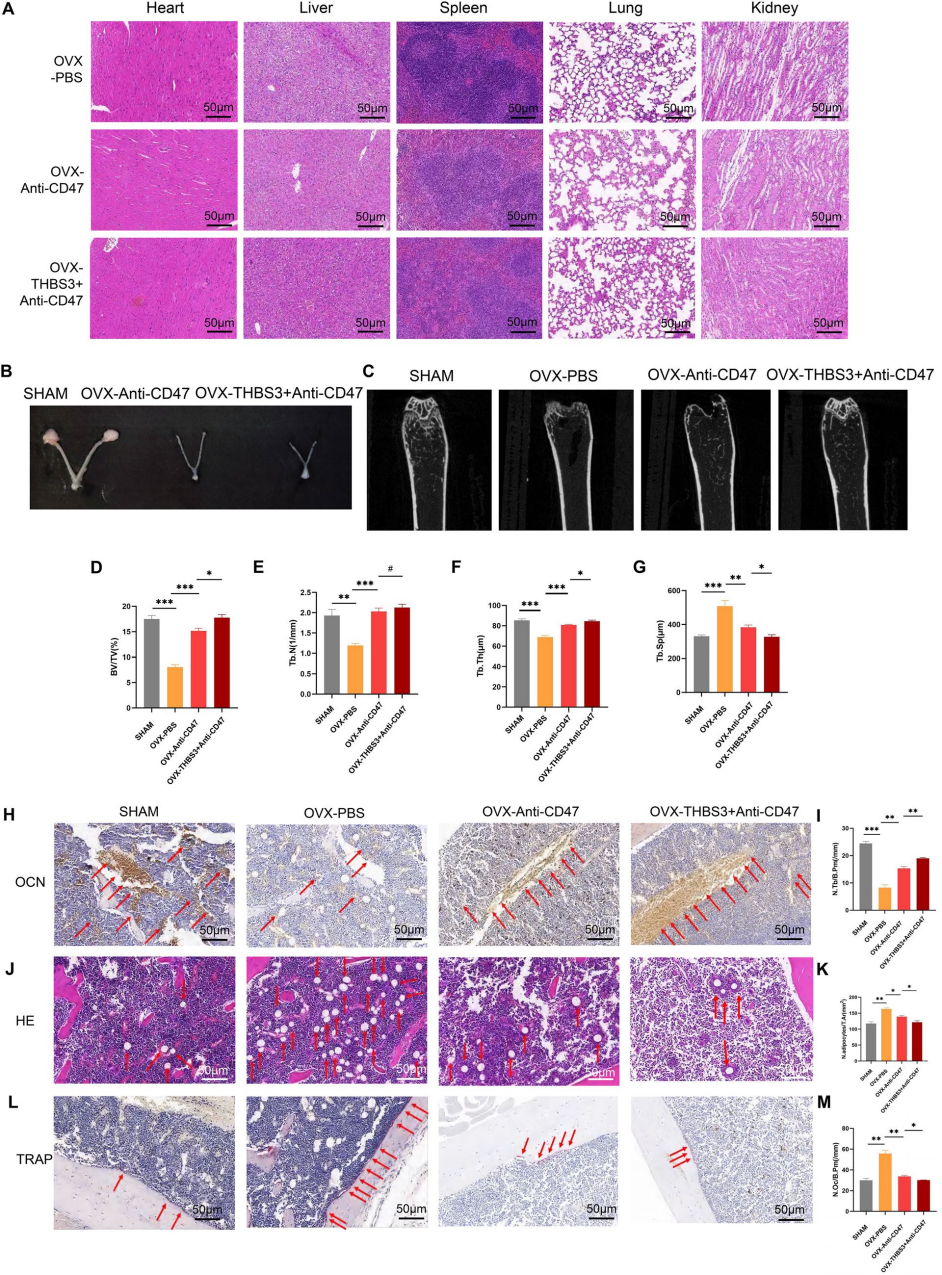
In vivo experiments to validate the effect of CD47 on the progression of osteoporosis in OVX mice. (A) HE staining of pathological sections of heart, liver, spleen, lungs and kidneys; (B) uterus of SHAM and OVX groups; (C) Micro CT of the distal femur; (D–G) Micro CT osteometric parameters; (H) OCN staining showed representative images of osteoblasts deposited on the bone surface; (I) quantitative analysis of the number of osteoblasts deposited on the bone surface versus the area ratio of the area; (J) HE staining showing the distribution of fat vacuoles in the bone marrow cavity of mice; (K) quantitative analysis of the ratio of the number of fat vacuoles in the bone marrow cavity to the area of the area; (L) osteoclasts deposited on the bone surface by TRAP staining; (M) quantitative analysis of the ratio of the number of osteoclasts deposited on the bone surface to the area of the region. *p < 0.05, **p < 0.01, ***p < 0.001.

**FIGURE 9:**
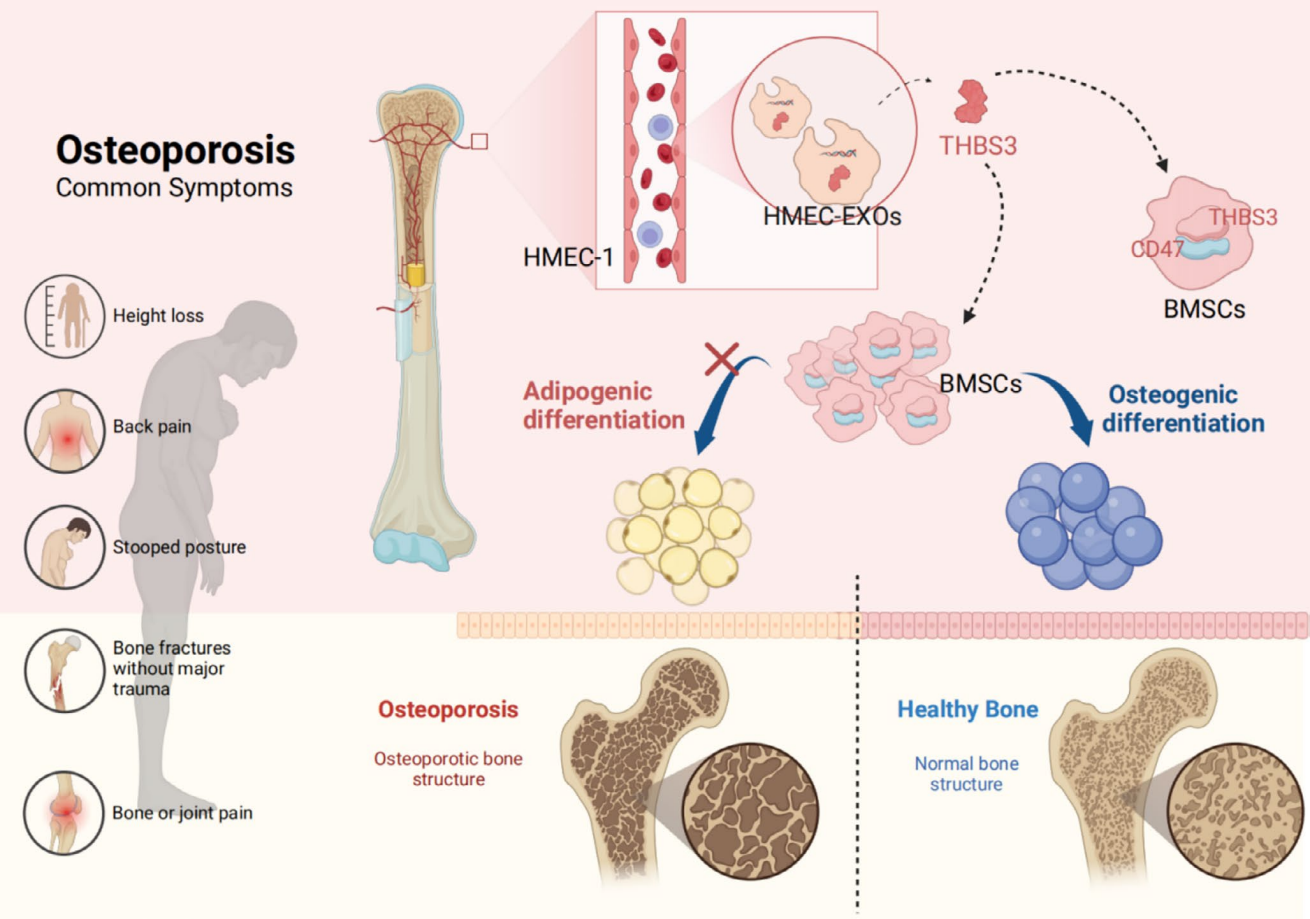
Schematic diagram of the mechanism EC‐EXOs‐derived THBS3‐CD47 axis to ameliorate osteoporosis. EC‐EXOs could regulate the differentiation of BMSCs through the key protein THBS3, which inhibits their differentiation to lipogenic cells and promotes their differentiation to osteoblasts. THBS3 could down‐regulate the expression of its downstream target CD47 and delay the process of bone loss in aged mice and OVX mice, which may provide a novel frontier for the treatment of osteoporosis.

The uterus of the sham‐operated group showed no obvious abnormality, and the uterus of the OVX‐CD47 antibody group and the OVX‐THBS3 + CD47 antibody group were significantly shrunken, implying that the OVX mice modelling was successful, and HE staining confirmed the biocompatibility across major organs (Figure 8A,B). Micro‐CT analysis of femurs revealed that CD47 antibody significantly enhanced cancellous bone formation (Figure 8C). Furthermore, the THBS3 + Anti‐CD47 group showed a greater increase in cancellous bone formation compared to the group treated with CD47 antibody alone (Figure 8C–G). OCN staining demonstrated a marked rise in osteoblast deposition on the cancellous bone surface in the Anti‐CD47 group (Figure 8H,I). This effect was further enhanced in the THBS3 + Anti‐CD47 group, indicating that THBS3 enhances the bone‐forming effects of CD47 antibody. HE and TRAP staining confirmed that CD47 antibody treatment reduced the number of adipocytes and osteoclasts (Figure 8J–M). Notably, these effects were significantly more pronounced in the THBS3 + Anti‐CD47 group. Taken together, these findings demonstrate that CD47 antibody can mitigate ageing‐related and osteoporosis‐associated bone loss by promoting osteoblast activity, reducing adipocyte accumulation, and suppressing osteoclast activity (Figure 9). Additionally, THBS3 synergistically enhances these effects, underscoring its potential as a complementary therapeutic strategy.

## 4 Discussion

Osteoporosis is a common systemic bone disease that leads to bone fragility and increased fracture risk. Further study of the bone marrow microenvironment is essential for developing strategies to reduce bone loss and slow the progression of osteoporosis. Emerging studies have reported the osteogenic role of exosomes. It has been found that BMSCs‐derived exosomes significantly promoted the osteogenic differentiation of BMSCs in vitro, while BMSCs‐specific aptamer functionalisation of BMSCs‐derived exosomes targeting bone efficiently promoted bone regeneration [21]. BMSCs‐derived exosomes could promote osteogenesis and angiogenesis [22]. In addition, exosome‐based bone‐targeted drug delivery can alleviate impaired bone formation and bone loss [23]. EC‐EXOs showed more effective bone targeting and suppressed osteoclast activity and osteoporosis [16]. Our previous studies demonstrated that EC‐EXOs can influence the course of osteoporosis by regulating the differentiation of BMSCs [20].

Exosomal proteins play an important role in exosome functioning. Nevertheless, the mechanism of action of how EC‐EXOs regulate key proteins to promote osteogenic differentiation of BMSCs remains ambiguous. Therefore, this study analysed from the perspective of proteomics to screen the key proteins in the process of EC‐EXOs affecting the differentiation of BMSCs to elucidate the molecular mechanism. The differentially expressed proteins in EC‐EXOs and HMEC‐1 samples were analysed by computational biology, and the expression level of THBS3 in EC‐EXOs was significantly higher compared to HMEC‐1.

The Thrombospondins family (THBS) consists of five secreted proteins that are widely distributed in the extracellular matrix of various tissues [24]. Different from others, THBS3 is a pentameric glycoprotein involved in regulating skeletal maturation and mediating cell‐to‐cell and cell‐to‐matrix interactions [25, 26, 27]. It has been demonstrated that THBS3 gene knockout, or combined THBS3/5/Col9 knockout, displayed trabecular bone abnormalities and impaired growth plate and long bone development in mice, highlighting the critical role of THBS3 in skeletal development [28, 29]. Dalla‐Torre et al. further confirmed that THBS3 is also associated with the development of osteosarcoma, maintaining angiogenesis and promoting tumour development [30]. Additionally, Costa and colleagues highlighted the involvement of THBS3 in regulating angiogenesis and vascular secretion [31]. Considering the results of computational biology, it was speculated that the key protein in regulating the differentiation of BMSCs by EC‐EXOs may be THBS3. RT‐qPCR experiments verified the results, and further study investigates whether EC‐EXOs affect BMSC differentiation through THBS3 in vivo and ex vivo models. Cellular experiments showed that THBS3 treatment enhanced the differentiation of BMSCs toward osteogenesis and inhibited the differentiation of BMSCs toward lipogenesis. In aged‐OVX models, THBS3 promoted cancellous bone formation by increasing the number of osteoblasts and decreasing the number of adipocytes and osteoclasts. Collectively, these results suggested that THBS3 delayed the progression of osteoporosis and plays a pivotal role in regulating the vascular‐osteogenic coupling crosstalk network in the bone marrow.

To further investigate the molecular mechanism of the THBS3 in the bone marrow microenvironment, we screened for downstream target genes (CD47, Itgb1 and Itga2b). CD47 is expressed on the membrane surface of almost all normal cells and is a highly glycosylated and widely expressed immunoglobulin superfamily protein involved in cell proliferation, adhesion, migration, apoptosis, and phagocytosis [32]. Xu et al. confirmed a direct link between CD47 and age‐related bone loss, elucidating a strong connection between immune surveillance and bone homeostasis [33]. They hypothesized that THBS3 regulates BMSC differentiation by targeting CD47. To validate this hypothesis, we treated BMSCs with THBS3 and observed a significant downregulation of CD47 expression, supporting the notion that THBS3 may exert its effects through the modulation of CD47.

CD47 could serve as a receptor for THBS1. The absence of the THBS1‐CD47 signalling pathway promoted the tissue‐specific and age‐dependent accumulation of normally functioning mitochondria, resulting in enhanced physical function, reduced reactive oxygen species production and improved metabolic efficiency [34]. Porpiglia et al. found that with muscle stem cells ageing, CD47 expression is significantly upregulated in an age‐dependent manner, exerting multiple negative effects on the regenerative capacity of ageing muscle [35]. Additionally, researchers reported that CD47 knockout mice exhibit intrinsic defects in osteoblast and osteoclast differentiation, highlighting the critical role of CD47 in the regulation of bone formation and resorption, and in the overall maintenance of the skeletal system [36]. To further investigate the underlying mechanism, we examined whether the effects of CD47 antibody on the bone marrow microenvironment are mediated through the THBS3‐CD47 axis by utilising aged and OVX models. In vivo experiments demonstrated that treatment with CD47 antibody reduced the number of adipocytes and osteoclasts in both aged and OVX mice. Furthermore, the combined treatment of THBS3 with CD47 antibody resulted in a significantly greater reduction in adipocytes and osteoclasts compared to treatment with CD47 antibody alone. Taken together, the CD47 antibody effectively delays ageing processes in aged mice and inhibits osteoporosis progression in OVX mice, highlighting a potential approach for targeting age‐related bone diseases. THBS3 enhances the therapeutic efficacy of CD47 antibody through a synergistic interaction. The therapeutic advantages of targeting CD47 are prominently emphasised by its unique regulatory mechanisms, which differ significantly from those of other immune checkpoints in the context of bone remodelling. The SIRPα‐CD47 axis mainly exerts its influence on osteoclast activity via phagocytic signalling [37]. However, our research found that the EC‐EXOs/THBS3‐CD47 axis principally governs the differentiation of BMSCs. This represents a crucial divergence in strategies for promoting bone formation. Significantly, immune checkpoints like PD‐1/PD‐L1 impact bone homeostasis in an indirect manner. They achieve this by modulating inflammatory responses and activating osteoclasts [38], a process that may potentially bring about systemic immunomodulatory risks. Conversely, CD47 plays a dual‐function role in skeletal biology.

While this study clearly demonstrates the regulatory role of THBS3 through CD47‐mediated signalling, several limitations call for further in‐depth investigation. Firstly, alternative receptors like CD36 and integrins might be involved in the regulatory network of THBS3. Emerging evidence indicated that CD36 interacted with thrombospondin family proteins [39] and competed with CD47 for THBS binding in the context of bone metabolism [40, 41]. In particular, the crosstalk between CD36 and CD47 has been established in the regulation of osteoclasts [41], suggesting potential compensatory mechanisms in the differentiation of BMSCs. Secondly, the TGF‐β and Wnt pathways, both of which are crucial for skeletal development, may interact with THBS3 signalling. Low‐dose TGF‐β1 promotes BMSC proliferation by activating the Wnt/β‐catenin pathway [42], while the inhibition of Wnt3a/β‐catenin mediates the anti‐fibrotic effects of BMSCs [43]. These findings imply possible cross‐talk between THBS3‐CD47 signalling and these conserved pathways, which necessitates systematic exploration.

In comparison with well‐established osteogenic regulators, THBS3 exhibits distinct mechanistic characteristics. Whereas BMP‐2 mainly activates Smad‐dependent cascades to induce osteogenesis [44, 45] and Runx2 functions as a master transcriptional regulator [46], THBS3 modulates differentiation through CD47‐mediated membrane signalling with unique downstream effectors. Notably, our study disclosed the bidirectional regulatory capacity of THBS3, that is the enhancing osteogenesis while suppressing adipogenesis, a feature not commonly seen in canonical BMP signalling. This functional divergence might originate from the extracellular matrix localisation of THBS3 and its ability to integrate mechanical signals with biochemical pathways, in contrast to the soluble factor nature of BMP‐2. Future research should systematically compare the temporal expression patterns and pathway crosstalk among these regulators to determine their hierarchical relationships in bone homeostasis.

## 5 Conclusion

Our study demonstrates that EC‐EXOs influence BMSC differentiation and mitigate bone loss in aged and OVX mice by regulating THBS3. Specifically, THBS3 derived from EC‐EXOs inhibits CD47 expression in BMSCs, thereby promoting osteogenesis. Through the THBS3‐CD47 axis, EC‐EXOs enhance the osteogenic differentiation of BMSCs and suppress adipogenic differentiation. In aged and OVX mice, EC‐EXOs increase osteoblast deposition on the bone surface, reduce fat vacuole accumulation in the bone marrow, and inhibit osteoclast activity via modulation of the THBS3‐CD47 signalling pathway. Neutralising antibodies targeting CD47 may be potential drugs for treating ageing‐associated bone loss. The subsequent clinical application of targeting CD47 requires further study.

## Author Contributions

Q.L. and R.X. provided direction and guidance throughout the preparation of this manuscript. J.W. and Z.Z. wrote and edited the manuscript. Q.L. reviewed and made significant revisions to the manuscript. Z.Z., W.C., Y.C., Q.Z., Y.C., and Z.O. collected and prepared the related papers. All authors read and approved the final manuscript.

## Ethics Statement

The animal experimental protocol was approved by the Experimental Animal Ethics Committee of the Second Xiangya Hospital of Central South University (ethical approval number: 20230489).

## Conflicts of Interest

The authors declare no conflicts of interest.

## Supporting information


**Data S1.** Supporting Information.

## Data Availability

The data that support the findings of this study are available on request from the corresponding author. The data are not publicly available due to privacy or ethical restrictions.

## References

[1] Y. Fang , J. Zhu , J. Fan , et al., “Dietary Inflammatory Index in Relation to Bone Mineral Density, Osteoporosis Risk and Fracture Risk: A Systematic Review and Meta‐Analysis,” Osteoporosis International: A Journal Established as Result of Cooperation Between the European Foundation for Osteoporosis and the National Osteoporosis Foundation of the USA 32 (2021): 633–643. 32740669 10.1007/s00198-020-05578-8

[2] P. Deng , Q. Yuan , Y. Cheng , et al., “Loss of KDM4B Exacerbates Bone‐Fat Imbalance and Mesenchymal Stromal Cell Exhaustion in Skeletal Aging,” Cell Stem Cell 28 (2021): 1057–1073.e1057. 33571444 10.1016/j.stem.2021.01.010 PMC8178178

[3] M. J. Favus , “Bisphosphonates for Osteoporosis,” New England Journal of Medicine 363 (2010): 2027–2035. 21083387 10.1056/NEJMct1004903

[4] M. E. Kraenzlin and C. Meier , “Parathyroid Hormone Analogues in the Treatment of Osteoporosis,” Nature Reviews. Endocrinology 7 (2011): 647–656. 10.1038/nrendo.2011.108 21750510

[5] W. Cui , X. Yang , Y. Dou , et al., “Effects of Tetrahedral DNA Nanostructures on the Treatment of Osteoporosis,” Cell Proliferation 57 (2024): e13625. 38414318 10.1111/cpr.13625 PMC11216938

[6] T. Rachner , S. Khosla , and L. Hofbauer , “Osteoporosis: Now and the Future,” Lancet (London, England) 377 (2011): 1276–1287. 21450337 10.1016/S0140-6736(10)62349-5 PMC3555696

[7] F. An , X. Wang , C. Wang , et al., “Research Progress on the Role of lncRNA‐miRNA Networks in Regulating Adipogenic and Osteogenic Differentiation of Bone Marrow Mesenchymal Stem Cells in Osteoporosis,” Front Endocrinol (Lausanne) 14 (2023): 1210627. 37645421 10.3389/fendo.2023.1210627 PMC10461560

[8] J. A. Cauley , “Osteoporosis: Fracture Epidemiology Update 2016,” Current Opinion in Rheumatology 29 (2017): 150–156. 28072591 10.1097/BOR.0000000000000365

[9] N. P. Hessvik and A. Llorente , “Current Knowledge on Exosome Biogenesis and Release,” Cellular and Molecular Life Sciences 75 (2018): 193–208. 28733901 10.1007/s00018-017-2595-9 PMC5756260

[10] F. Mehryab , S. Rabbani , S. Shahhosseini , et al., “Exosomes as a Next‐Generation Drug Delivery System: An Update on Drug Loading Approaches, Characterization, and Clinical Application Challenges,” Acta Biomaterialia 113 (2020): 42–62. 32622055 10.1016/j.actbio.2020.06.036

[11] R. Zuo , M. Liu , Y. Wang , et al., “BM‐MSC‐Derived Exosomes Alleviate Radiation‐Induced Bone Loss by Restoring the Function of Recipient BM‐MSCs and Activating Wnt/β‐Catenin Signaling,” Stem Cell Research & Therapy 10 (2019): 30. 30646958 10.1186/s13287-018-1121-9 PMC6334443

[12] X. Qi , J. Zhang , H. Yuan , et al., “Exosomes Secreted by Human‐Induced Pluripotent Stem Cell‐Derived Mesenchymal Stem Cells Repair Critical‐Sized Bone Defects Through Enhanced Angiogenesis and Osteogenesis in Osteoporotic Rats,” International Journal of Biological Sciences 12 (2016): 836–849. 27313497 10.7150/ijbs.14809 PMC4910602

[13] Y. Peng , S. Wu , Y. Li , and J. L. Crane , “Type H Blood Vessels in Bone Modeling and Remodeling,” Theranostics 10 (2020): 426–436. 31903130 10.7150/thno.34126 PMC6929606

[14] Z. Yang , X. Li , X. Gan , et al., “Hydrogel Armed With Bmp2 mRNA‐Enriched Exosomes Enhances Bone Regeneration,” Journal of Nanobiotechnology 21 (2023): 119. 37020301 10.1186/s12951-023-01871-w PMC10075167

[15] Y. Jin , M. Xu , H. Zhu , et al., “Therapeutic Effects of Bone Marrow Mesenchymal Stem Cells‐Derived Exosomes on Osteoarthritis,” Journal of Cellular and Molecular Medicine 25 (2021): 9281–9294. 34448527 10.1111/jcmm.16860 PMC8500984

[16] H. Song , X. Li , Z. Zhao , et al., “Reversal of Osteoporotic Activity by Endothelial Cell‐Secreted Bone Targeting and Biocompatible Exosomes,” Nano Letters 19 (2019): 3040–3048. 30968694 10.1021/acs.nanolett.9b00287

[17] R. Kalluri and V. S. LeBleu , “The Biology, Function, and Biomedical Applications of Exosomes,” Science 367, no. 6478 (2020): eaau6977. 32029601 10.1126/science.aau6977 PMC7717626

[18] D. K. W. Ocansey , L. Zhang , Y. Wang , et al., “Exosome‐Mediated Effects and Applications in Inflammatory Bowel Disease,” Biological Reviews of the Cambridge Philosophical Society 95 (2020): 1287–1307. 32410383 10.1111/brv.12608 PMC7540363

[19] K. Huang , S. Cai , T. Fu , et al., “Wnt10b Regulates Osteogenesis of Adipose‐Derived Stem Cells Through Wnt/β‐Catenin Signalling Pathway in Osteoporosis,” Cell Proliferation 57 (2024): e13522. 37340715 10.1111/cpr.13522 PMC10771102

[20] J. Wang , X. Xie , H. Li , et al., “Vascular Endothelial Cells‐Derived Exosomes Synergize With Curcumin to Prevent Osteoporosis Development,” iScience 27 (2024): 109608. 38623340 10.1016/j.isci.2024.109608 PMC11016789

[21] Z. W. Luo , F. X. Li , Y. W. Liu , et al., “Aptamer‐Functionalized Exosomes From Bone Marrow Stromal Cells Target Bone to Promote Bone Regeneration,” Nanoscale 11 (2019): 20884–20892. 31660556 10.1039/c9nr02791b

[22] G. D. Lu , P. Cheng , T. Liu , and Z. Wang , “BMSC‐Derived Exosomal miR‐29a Promotes Angiogenesis and Osteogenesis,” Frontiers in Cell and Development Biology 8 (2020): 608521. 10.3389/fcell.2020.608521 PMC7755650 33363169

[23] J. Guo , F. Wang , Y. Hu , et al., “Exosome‐Based Bone‐Targeting Drug Delivery Alleviates Impaired Osteoblastic Bone Formation and Bone Loss in Inflammatory Bowel Diseases,” Cell Reports Medicine 4 (2023): 100881. 36603578 10.1016/j.xcrm.2022.100881 PMC9873828

[24] J. C. Adams and J. Lawler , “The Thrombospondins,” Cold Spring Harbor Perspectives in Biology 3 (2011): a009712. 21875984 10.1101/cshperspect.a009712 PMC3179333

[25] Y. Lu , X. Kong , W. Zhong , M. Hu , and C. Li , “Diagnostic, Therapeutic, and Prognostic Value of the Thrombospondin Family in Gastric Cancer,” Frontiers in Molecular Biosciences 8 (2021): 647095. 33996903 10.3389/fmolb.2021.647095 PMC8113821

[26] E. G. Frolova , J. Drazba , I. Krukovets , et al., “Control of Organization and Function of Muscle and Tendon by Thrombospondin‐4,” Matrix Biology 37 (2014): 35–48. 24589453 10.1016/j.matbio.2014.02.003 PMC4150858

[27] L. Carminati and G. Taraboletti , “Thrombospondins in Bone Remodeling and Metastatic Bone Disease,” American Journal of Physiology. Cell Physiology 319 (2020): C980–c990. 32936697 10.1152/ajpcell.00383.2020

[28] K. D. Hankenson , S. G. Hormuzdi , J. A. Meganck , and P. Bornstein , “Mice With a Disruption of the Thrombospondin 3 Gene Differ in Geometric and Biomechanical Properties of Bone and Have Accelerated Development of the Femoral Head,” Molecular and Cellular Biology 25 (2005): 5599–5606. 15964815 10.1128/MCB.25.13.5599-5606.2005 PMC1156967

[29] K. Tan and J. Lawler , “The Interaction of Thrombospondins With Extracellular Matrix Proteins,” Journal of Cell Communication and Signaling 3 (2009): 177–187. 19830595 10.1007/s12079-009-0074-2 PMC2778591

[30] C. A. Dalla‐Torre , M. Yoshimoto , C. H. Lee , et al., “Effects of THBS3, SPARC and SPP1 Expression on Biological Behavior and Survival in Patients With Osteosarcoma,” BMC Cancer 6 (2006): 237. 17022822 10.1186/1471-2407-6-237 PMC1609181

[31] A. Costa , J. Afonso , C. Osório , et al., “miR‐363‐5p Regulates Endothelial Cell Properties and Their Communication With Hematopoietic Precursor Cells,” Journal of Hematology & Oncology 6 (2013): 87. 24257019 10.1186/1756-8722-6-87 PMC3874849

[32] R. Bouwstra , T. van Meerten , and E. Bremer , “CD47‐SIRPα Blocking‐Based Immunotherapy: Current and Prospective Therapeutic Strategies,” Clinical and Translational Medicine 12, no. 8 (2022): e943, 10.1002/ctm2.943. 35908284 PMC9339239

[33] R. Xu , H. Xie , X. Shen , et al., “Impaired Efferocytosis Enables Apoptotic Osteoblasts to Escape Osteoimmune Surveillance During Aging,” Advanced Science (Weinheim) 10 (2023): e2303946. 10.1002/advs.202303946 PMC10754079 37897313

[34] E. P. Frazier , J. S. Isenberg , S. Shiva , et al., “Age‐Dependent Regulation of Skeletal Muscle Mitochondria by the Thrombospondin‐1 Receptor CD47,” Matrix Biology 30 (2011): 154–161. 21256215 10.1016/j.matbio.2010.12.004 PMC3070423

[35] E. Porpiglia , T. Mai , P. Kraft , et al., “Elevated CD47 Is a Hallmark of Dysfunctional Aged Muscle Stem Cells That Can Be Targeted to Augment Regeneration,” Cell Stem Cell 29 (2022): 1653–1668.e1658. 36384141 10.1016/j.stem.2022.10.009 PMC9746883

[36] L. A. Maile , V. E. DeMambro , C. Wai , et al., “An Essential Role for the Association of CD47 to SHPS‐1 in Skeletal Remodeling,” Journal of Bone and Mineral Research 26 (2011): 2068–2081. 21638321 10.1002/jbmr.441 PMC3383326

[37] Y. Murata , T. Kotani , H. Ohnishi , and T. Matozaki , “The CD47‐SIRPα Signalling System: Its Physiological Roles and Therapeutic Application,” Journal of Biochemistry 155 (2014): 335–344. 24627525 10.1093/jb/mvu017

[38] J. Tang , “Immune Checkpoint Inhibitors: Friend or Foe for Osteoporosis,” Therapeutic Advances in Endocrinology and Metabolism 14 (2023): 20420188231157194. 36876151 10.1177/20420188231157194 PMC9983083

[39] L. Xia , Z. Zhou , X. Chen , et al., “Ligand‐Dependent CD36 Functions in Cancer Progression, Metastasis, Immune Response, and Drug Resistance,” Biomedicine & Pharmacotherapy 168 (2023): 115834. 37931517 10.1016/j.biopha.2023.115834

[40] W. Choi , A. K. Nensel , S. Droho , et al., “Thrombospondin‐1 Proteomimetic Polymers Exhibit Anti‐Angiogenic Activity in a Neovascular Age‐Related Macular Degeneration Mouse Model,” Science Advances 9 (2023): eadi8534. 37831763 10.1126/sciadv.adi8534 PMC10575579

[41] S. V. Koduru , B. H. Sun , J. M. Walker , et al., “The Contribution of Cross‐Talk Between the Cell‐Surface Proteins CD36 and CD47‐TSP‐1 in Osteoclast Formation and Function,” Journal of Biological Chemistry 293 (2018): 15055–15069. 30082316 10.1074/jbc.RA117.000633 PMC6166722

[42] F. Zhang , T. Ren , J. Wu , and J. Niu , “Small Concentrations of TGF‐β1 Promote Proliferation of Bone Marrow‐Derived Mesenchymal Stem Cells via Activation of Wnt/β‐Catenin Pathway,” Indian Journal of Experimental Biology 53 (2015): 508–513. 26349313

[43] Z. Liu , S. Zhou , Y. Zhang , and M. Zhao , “Rat Bone Marrow Mesenchymal Stem Cells (BMSCs) Inhibit Liver Fibrosis by Activating GSK3β and Inhibiting the Wnt3a/β‐Catenin Pathway,” Infectious Agents and Cancer 17 (2022): 17. 35440002 10.1186/s13027-022-00432-4 PMC9017036

[44] H. Cai , J. Zou , W. Wang , and A. Yang , “BMP2 Induces hMSC Osteogenesis and Matrix Remodeling,” Molecular Medicine Reports 23, no. 2 (2021): 125, 10.3892/mmr.2020.11764. 33300084 PMC7751477

[45] X. J. Chen , Y. S. Shen , M. C. He , et al., “Polydatin Promotes the Osteogenic Differentiation of Human Bone Mesenchymal Stem Cells by Activating the BMP2‐Wnt/β‐Catenin Signaling Pathway,” Biomedicine & Pharmacotherapy 112 (2019): 108746. 30970530 10.1016/j.biopha.2019.108746

[46] L. Deng , G. Hu , L. Jin , C. Wang , and H. Niu , “Involvement of microRNA‐23b in TNF‐α‐Reduced BMSC Osteogenic Differentiation via Targeting runx2,” Journal of Bone and Mineral Metabolism 36 (2018): 648–660. 29234953 10.1007/s00774-017-0886-8

