# Supplementary material for: Mechanism of EC‐EXOs‐Derived THBS3 Targeting CD47 to Regulate BMSCs Differentiation to Ameliorate Bone Loss
Source: Cell Prolif. 2025 Jun 13;58(12):e70066. doi: 10.1111/cpr.70066 (PMC12686127; doi:10.1111/cpr.70066)
Supplement: Supplementary file 1 — Data S1. Supporting Information. [file CPR-58-e70066-s001.docx]

# 1 Supplementary methods and materials

# 1.1 Experimental materials and reagents

Human Microvascular Endothelial Cell (HMEC-1) was purchased from Shanghai Zhongqiao Xinzhou Biotechnology Co., Ltd, and the culture medium was MCDB131 containing 10% fetal bovine serum.

Bone Marrow Stem Cells (BMSCs) were extracted from 4-week-old C57BL/6J mice, and the culture medium was α-MEM containing 15% fetal bovine serum.

1. week-old C57BL/6J mice, 8-week-old female C57BL/6J mice, and 12-month-old male C57BL/6J mice (production licence: SCXK Xiang 2016-0002). The main reagents of the experiment (Table S1) are listed below:

Table S1. The main reagents of this study.

| **REAGENT or RESOURCE** | **SOURCE** |
| --- | --- |
| MCDB131 medium | Procell（China） |
| Exosome-free serum | Biological Industries（Israel） |
| Exosome extraction kit (EXO-Quick-TC™ Tissue culture Media Exosomes precipitation solution) | System Biosciences（USA) |
| PBS | Procell（China） |
| Fetal bovine serum | CellMax（China） |
| α-MEM medium | CellMax（China） |
| 0.25% EDTA Trypsin solution | Procell（China） |
| Penicillin-Streptomycin Dual Antibody | Procell（China） |
| Anti-CD9 Antibody | Abcam（UK） |
| Anti-CD63 Antibody | Abcam（UK） |
| Anti-TSG101 Antibody | Abcam（UK） |
| Anti-Calnexin Antibody | Cell Signaling Technology（USA） |
| Horseradish peroxidase labelled goat anti-rabbit IgG (H+L) | Beyotime（China） |
| Horseradish peroxidase labelled goat anti-mouse IgG (H+L) | Beyotime（China） |
| Western and IP cell lysates | Beyotime（China） |
| PMSF | Beyotime（China） |
| Pierce BCA Protein assay Kit | Thermo Fisher Scientific（USA） |
| PageRulerTM Prestained Protein Ladder | Thermo Fisher Scientific（USA） |
| LumiBest Premier ECL Luminous Liquid | Share-bio（China） |
| 4% paraformaldehyde | Sinopharm Chemical Reagent（China） |
| PKH67 Staining Kit | Sigma-Aldrich（USA） |
| Vector Anti-Fluorescence Extraction Sealer | Vector labs（USA） |
| Insulin | Procell（China） |
| Dexamethasone (DEX) | Sigma-Aldrich（USA） |
| Vitamin C | Sigma-Aldrich（USA） |
| Indomethacin | Sigma-Aldrich（USA） |
| 3-Isobutyl-1-methylxanthine (IBMX) | Sigma-Aldrich（USA） |
| Beta-glycerophosphate (BGP) | Sigma-Aldrich（USA） |
| Chlorohexadecylpyridine (CPC) | Sigma-Aldrich（USA） |
| Anhydrous Ethanol | Anhui Jingyueguan New Material Technology Co.（China） |
| Methanol | Anhui Jingyueguan New Material Technology Co.（China） |
| Isopropyl alcohol | Anhui Jingyueguan New Material Technology Co.（China） |
| Chloroform | Anhui Jingyueguan New Material Technology Co.（China） |
| DEPC water | Biosharp（China） |
| RNAiso plus | TakaRa（Japan） |
| ChamQ Universal SYBR qPCR Master Mix | Vazyme（China） |
| HiScript® II Q RT SuperMix for qPCR (+g DNA wiper) | Vazyme（China） |
| 4% paraformaldehyde | Biosharp（China） |
| Alizarin Red Staining Solution | Starfish Biotechnology (Suzhou) Co. |
| Oil Red O Stain | Starfish Biotechnology (Suzhou) Co. |
| Na2HPO4 | Sigma-Aldrich (USA) |
| Hydrochloric acid | Anhui Jinyueguan New Material Technology Co Ltd (China) |
| Dimethyl sulfoxide (DMSO) | GBCBIO Technologies (China) |
| Sodium chloride | Sinopharm Chemical Reagent (China) |
| Na2HPO4-12H2O | Sinopharm Chemical Reagent (China) |
| NaH2PO4-2H2O | Sinopharm Chemical Reagent (China) |
| Disodium EDTA | Sinopharm Chemical Reagent (China) |
| Sodium hydroxide | Sinopharm Chemical Reagent (China) |
| Citric acid monohydrate | Sinopharm Chemical Reagents (China) |
| BSA | Solarbio (China) |
| Xylene | Sinopharm Chemical Reagent (China) |
| n-Butanol | Sinopharm Chemical Reagent (China) |
| Ammonium hydroxide | Sinopharm Chemical Reagent (China) |
| Neutral gum | Sinopharm Chemical Reagent (China) |
| 3% Hydrogen Peroxide | Sinopharm Chemical Reagent (China) |
| HE Dyeing Solution Set | Wuhan Google Biotechnology (China) |
| HE Dyeing Solution | Servicebio（China） |
| Differentiation solution | Servicebio（China） |
| Blue Return Liquid | Servicebio（China） |
| Eco-friendly Dewaxing Clear Solution | Servicebio（China） |
| TRAP Dyeing Solution Set | Servicebio（China） |
| ALP Stain Kit | Servicebio（China） |
| Recombinant Human Thrombospondin-3 Protein (THBS3) | Biotechne（USA） |
| InVivoMAb anti-mouse CD47 (IAP) | BioXcell（USA） |
| Gastric Enzyme | Servicebio（China） |

**1.2 Western blot (WB)**

Take 25 μL each of standard and diluted EC-EXOs samples to be tested (3 μL exosomes + 27 μL PBS) and add them into 96-well plate; take 200 μL of BCA working solution in 96-well plate and incubate at 37° for 30 min; the absorbance of A562 was measured by enzyme marker; the standard curve was plotted according to the measured results of the standard. protein concentration.

Dilute the sample to the desired concentration and add loading buffer (4:1). Mix thoroughly and centrifuge, place at 95°C, heat for 5min, and cool in an ice box. The samples were centrifuged at 12000×g for 5min, and added to the spiked wells (including marker) in order. Turn on the power, electrophoresis at constant voltage 80 V for 30 min, then continue electrophoresis at constant voltage 120 V for 60 to 90 min. Activate the PVDF membrane using methanol (3 min), equilibrate the PVDF membrane with filter paper in buffer for 5 min, and immerse it together with the gel sandwich in a tray with membrane transfer solution. The gel was removed and the separating gel was rinsed once with 1× transmembrane buffer. The ‘sandwich’ was formed in the form of ‘sponge - three-layer filter paper - separation gel - PVDF membrane - three-layer filter paper - sponge’, and the membrane was transferred on ice at 260 mA for 90 min. After membrane transfer, the PVDF membrane was placed in an incubator with 5% skimmed milk and TBST, and incubated for 1h at room temperature on a decolourising shaker with shaking. After sealing was completed, the membrane was placed face up and washed 3 times at room temperature using TBST with shaking for 10min each time. The PVDF membrane was incubated in primary antibody dilution, shaken overnight at 4°C on a decolourising shaker and placed at room temperature for 3 shaking washes using TBST for 10min each. The secondary antibody of the corresponding species was added in 5% skimmed milk at the recommended ratio, and the membrane was incubated in a slow shaker at room temperature for 1h, and then washed 3 times at room temperature using TBST with shaking for 10min each time. Liquid A and liquid B in the ECL developing solution were prepared as working solution according to 1:1, and the membrane was placed in the imager to add ECL luminescent solution for chemiluminescence reaction, and photographs were taken. The protein bands were analysed using Image J image analysis software, and the ratio of the grey value of the target protein to that of the internal reference was taken as the relative expression of the target protein.

**1.3 Configuration of osteogenic and lipogenic induction solution**

Weigh about 88.06mg of vitamin C dissolved in 10ml PBS to make a vitamin C stock solution with a concentration of 0.05M, and pass through 0.22μM PES filter membrane to remove bacteria; About 2.1604g BGP and 3.9246mg DEX were weighed and dissolved in 10ml PBS to make a BGP stock solution with a concentration of 1M and a DEX stock solution with a concentration of 1mM, respectively, and were decontaminated by passing through a 0.22μM PES filter membrane; Weighed about 894.475mg of indomethacin dissolved in 10ml DMSO to make a stock solution of indomethacin at a concentration of 250mM; Weighing and measuring about 444.48mg of IBMX dissolved in 10ml DMSO to make IBMX stock solution with a concentration of 0.2M; Dispense the above stock solution and store it at -20℃ for spare use.

Sequentially add 7.5ml of fetal bovine serum, 500μl of double antibiotic, 50μl of 0.05mM vitamin C stock solution, 5μl of 1mM DEX stock solution, 500μl of 1M BGP stock solution into 50ml centrifuge tube, and then add α-MEM to 50ml. The complete medium for osteogenic induction containing 15% fetal bovine serum, 1% double antibody, 0.1nM DEX, 10mM BGP and 0.05mM vitamin C was prepared. 1 ml of insulin solution at a concentration of 10 mg/ml was diluted to 4 mg/ml with 1.5 ml of PBS and stored at 4°C to avoid repeated freezing and thawing; Sequentially add 7.5 ml of foetal bovine serum, 500 μl of double antibody, 50 μl of 0.05 M of vitamin C stock solution, 50 μl of 1 mM of DEX stock solution, 10 μl of 250 mM of indomethacin stock solution, 125 μl of 0.2 M of IBMX stock solution, and 62.5 μl of insulin at 4 mg/ml in 50 ml centrifugal tube, and add α-MEM to 50 ml. A complete medium for lipogenic induction containing 15% fetal bovine serum, 1% double antibody, 1 nM DEX, 0.05 mM vitamin C, 50 μM indomethacin, 0.5 mM IBMX and 5 μg/ml insulin can be prepared.

**1.4 RNA extraction**

Discard the cell culture medium in the six-well plate, and wash with PBS for 2-3 times; Aspirate the PBS, add 1ml of RNAex (Trizol) to each well, blow the cells repeatedly with a pipette gun (cell lysis can be clearly seen), and then transfer it to the ep tube; Let it stand for 5min at room temperature, add 200ul chloroform, mix well and let it stand for 5min; 12000g 4℃ centrifugation for 15min, take 400ul supernatant to a new ep tube, add 400ul isopropanol, mix gently and leave at room temperature for 10min; 12000g 4℃ centrifugation for 10min, discard the supernatant, add 1ml of 75% ethanol, wash the RNA precipitate (75% ethanol with DEPC water ready to use); 7500g 4 ℃ centrifugation 5min, discard the supernatant, dry 5min, add 20ul DEPC water to dissolve RNA, need to be blown uniformly (can be inverted ep tube on the paper for drying, if the ep tube after 5min the inner wall of the liquid is still left, the gun tip can be used to carefully suction away, pay attention to do not suction away from the precipitate, suction is not clean will reduce the concentration of RNA); Perform RNA concentration determination and store the RNA in -80℃ refrigerator.

**1.5 RT-qPCR**

***1.5.1 mRNA reverse transcription***

**Removal of genomic DNA** Remove RNA from -80℃ refrigerator, dissolve on ice, vortex centrifugation, so that the RNA is sufficiently mixed; prepare the following mixture in RNase-free centrifuge tubes (Table S2); good offices of the reaction system and centrifugation to mix uniformly; in the PCR instrument, incubate at 42℃ for 2 min .

Table S2. Reagents in RNase-free centrifuge tubes.

| **reagents** | **Amount of reagent added** |
| --- | --- |
| RNase-free ddH2O | to 16 ul |
| 4×gDNA wiper Mix | 4 ul |
| Template RNA | Total RNA：1 pg - 1 µg |

**Preparation of the reverse transcription reaction system** Add 5 × HiScript II qRT SuperMix II (4 ul) directly into the reaction tube according to the table below; mix well by centrifuging the reaction system.

**Perform reverse transcription reaction** In the PCR instrument, the reaction was firstly reacted at 50℃ for 15min, and then at 85℃ for 5 seconds to terminate the reaction. The product can be used immediately for qPCR reaction or stored at -20℃ and used within half a year; long-term storage is recommended to be stored at -70℃ after dispensing. cDNA should be avoided from repeated freeze-thawing.

**1.5.2 qPCR**

Prepare the following mixture in a qPCR tube (Table S3):

Table S3. Reagents in a qPCR tube.

| **Component** | **Volume** |
| --- | --- |
| 2 × ChamQ Universal SYBR qPCR Master Mix | 10.0 ul |
| Primer 1 (10 µM) | 0.4 ul |
| Primer 2 (10 µM) | 0.4 ul |
| Template DNA/cDNA | x ul |
| ddH2O | to 20.0 ul |

The qPCR reaction (Table S4) was performed under the following conditions:

Table S4. qPCR reaction steps.

| **Step** | | **Cycle** | **Temperature** | **Time** |
| --- | --- | --- | --- | --- |
| Stage 1 | Pre-denaturation | Rep：1 | 95℃ | 30 sec |
| Stage 2 | Cycling | Reps：40 | 95℃ | 3-10 sec |
|  |  |  | 60℃ | 10-30 sec |
| Stage 3 | Melting Curve | Use the instrument's default melting curve acquisition programme. | | |

Table S5. The primers for the relevant genes required for the experiment.

| **Primer name** | **Sequence (5′ to 3′)** | |
| --- | --- | --- |
| β-actin | Forward | GGCTGTATTCCCCTCCATCG |
|  | Reverse | CCAGTTGGTAACAATGCCATGT |
| OPN | Forward | ACCATGCAGAGAGCGAGGATT |
|  | Reverse | GGGACATCGACTGTAGGGACG |
| RUNX2 | Forward | AGTTCCCAAGCTTTCATC |
|  | Reverse | GGCAGGTAGGTGTGGTAGT |
| Osterix | Forward | CTACCCATCTGACTTTGCTC |
|  | Reverse | CACTATTTCCCACTGCCTT |
| OCN | Forward | ACTCTTGCCTCGTCCACT |
|  | Reverse | GGTCTCTTCACTACCTCGCT |
| mPparg | Forward | ATGGTTGACACAGAGATGC |
|  | Reverse | GAATGCGAGTGGTCTTCC |
| CD36 | Forward | CGATTAACATAAGTAAAGTTGCCATA |
|  | Reverse | CGCAGTGACTTTCCCAATAGGAC |
| Cebpα | Forward | TGGACAAGAACAGCAACGAG |
|  | Reverse | TCACTGGTCAACTCCAGCAC |
| P16 | Forward | ACATCAAGACATCGTGCGATATT |
|  | Reverse | CCAGCGGTACACAAAGACCA |
| Caspase3 | Forward | ATGGAGAACAACAAAACCTCAGT |
|  | Reverse | TTGCTCCCATGTATGGTCTTTAC |

**1.6 Animal experimental model construction**

Aged mouse model: adaptively feed 15-month-old male C57BL/6J mice for one week, then tail vein injection experiments can be carried out. OVX mouse osteoporosis model: 8-week-old female C57BL/6J mice were subjected to ovary removal surgery. The mice were anaesthetised, the limbs were fixed with the backs facing upwards, the surgical area (thigh root about 1 cm upwards) was sterilised with alcohol, a small longitudinal incision was made on the back with ophthalmic scissors, and the skin of the mice was bluntly separated from the subcutaneous tissue with forceps. Both sides were ligated approximately at the upper edge of the lumbar off-white triangle, where the red dots (ovaries) were visible, and the ovaries were excised and the wounds were closed with absorbable sutures. In the sham-operated group, only a piece of fat near the ovary was removed, and the rest of the surgical procedures were performed in the same way. After surgery, the mice were placed on an electric blanket to keep them warm and injected with appropriate saline to accelerate the recovery from anaesthesia.

**1.7 Method of making paraffin sections of bone tissue**

**Sampling:** the femur was fixed in tissue fixative overnight. Excess tissue around the femur was removed and trimmed flat, and the trimmed femur was placed in a dehydration box. **Dehydration:** the dehydration box is placed in a hanging basket and gradient alcohol dehydration and wax dipping are carried out sequentially in a dehydrator. **Embedding:** the wax-dipped femur is embedded. Pour the melted wax into the embedding frame, and quickly put the above femur specimens into the embedding frame according to the embedding surface and labelled well. Cool the wax block at -20℃ on the freezing table and trim the wax block after cooling and solidification. **Slicing:** the wax block was placed in a paraffin slicer with a slice thickness of 3 μm. the cut slices were spread on a spreader with 40°C warm water, and then the tissue was fished up with a slide and baked in an oven at 60°C. The slices were then cut to the thickness of 3 μm and then sliced into the slices.

**1.8 HE staining experiment for bone tissue sections**

**Dewaxing:** use xylene Ⅰ to soak baked paraffin section samples for 20min; use xylene Ⅱ to soak samples for 20min; when the wax is dissolved, soak samples in anhydrous ethanol Ⅰ for 5min; use anhydrous ethanol Ⅱ to soak samples for 5min; use anhydrous ethanol to brush and wash for 20s; after that, the alcohol will be rinsed clean. **Staining and dehydration:** HE staining solution 1 stained the sections for 3-5min, removed and washed until the sections were colourless; HE staining solution 2 stained the sections for 3-5s, and washed quickly with water; HE staining solution 3 stained sections in 3-5s, rapid water washing; Sections were sequentially soaked in 85% ethanol, 95% ethanol, HE staining solution 4, anhydrous ethanol I, anhydrous ethanol II, anhydrous ethanol III, n-butanol, xylene I, and xylene II for 3-5min respectively. **Sealing:** Sections were blown dry quickly and sealed with neutral gum.

Table S6. The specific composition of the dye solution.

| **Staining solution** | **Main ingredient** | **Storage condition** |
| --- | --- | --- |
| Stain 1 | Hematoxylin | RT |
| Stain 2 | Differentiation solution | RT |
| Stain 3 | Rebluing solution | RT |
| Stain 4 | Eosin | RT |

**1.9 TRAP staining experiments on bone tissue sections**

**De-waxing**. **TRAP incubation solution preparation:** dissolve 20mg of TRAP staining powder4 with 1mL of TRAP staining solution5 and mark it as A solution for spare (A solution should be configured at least 3 days in advance). TRAP incubation solution preparation: TRAP staining solution 1: TRAP staining solution 2: TRAP staining solution 3: Liquid A was mixed sequentially, then 0.282g TRAP staining powder 6 was added, fully dissolved and filtered for use, i.e. TRAP incubation solution. **Staining:** draw a circle with a histochemical pen and put the section into a wet box to be stained; use a rubber-tipped buret to suck up the prepared TRAP pre-incubation solution, and add it onto the tissue stained with the circle for 2-3h; Pour off the staining solution on the section, add the filtered TRAP incubation solution dropwise, and incubate for 20-30min; pour off the staining solution, and stain the section into TRAP staining solution 7 for 30s; Remove the slices and wash until the slices are colourless; put the slices into TRAP Staining Solution 8 for 3-5s and wash them quickly; put the slices into TRAP Staining Solution 9 for 3-5s and wash them quickly. **Dehydration:** the sections were dehydrated in anhydrous ethanol I for 5 min; transferred to anhydrous ethanol II for 5 min; then transferred to anhydrous ethanol III for 5 min. **Sealing:** the sections were put into xylene I for 5min; transferred to xylene II for 5min, and then put into the air vent to blow dry quickly, and then sealed with neutral gum.

Table S7. The specific composition of the dye solution.

| **Staining solution** | **Main ingredient** | **Storage condition** |
| --- | --- | --- |
| Stain 1 | Sodium nitrite | 4℃ |
| Stain 2 | Magenta hydrochloride | 4℃ |
| Stain 3 | buffer | 4℃ |
| Stain 4 | Naphthol | 4℃ |
| Stain 5 | DMF | 4℃ |
| Stain 6 | Tartaric acid | 4℃ |
| Stain 7 | Hematoxylin | RT |
| Stain 8 | Differentiation solution | RT |
| Stain 9 | Rebluing solution | RT |

**1.10 OCN immunohistochemical staining experiment on bone tissue sections**

**De-waxing. Antigen repair:** the repair solution is 1× citric acid (PH6.0), high temperature and high pressure repair, place the sections in an autoclave, add the repair solution, time it for 2min, then cool it down to room temperature, the repair is completed. **Endogenous enzyme blocking:** the sections were incubated in 3% H2O2 at room temperature for 20 min. washed with PBS 3 times for 5 min each time. **Serum closure:** draw a circle around the tissue with a histochemical pen, add drops of serum on the tissue and incubate for 30min at 37°C. Closure was performed with serum from the same source as the secondary antibody (e.g. 10% goat serum). **Primary antibody incubation:** dilute the antibody using primary antibody diluent, remove the serum from the slice, add antibody working solution dropwise on the tissue and incubate at 4℃ overnight. **Secondary antibody incubation (using HRP enzyme-labelled secondary antibody):** Prepare secondary antibody with PBST, add secondary antibody dropwise on the tissue, incubate at 37℃ for 1h, and wash with PBST for 3 times, 5min each time. **DAB colour development:** DAB working solution was added dropwise on the tissue, and when specific brown expression appeared on the slide, rinse off the DAB colour development solution and soak the slide in water. **Re-staining hematoxylin, dehydration, sealing:** the slide to the hematoxylin staining solution, staining 3-5min, rinse off the excess hematoxylin staining solution, after the nuclei become blue, placed in 0.5% hydrochloric acid alcohol differentiation solution to differentiate for 1-2s, and then put into the water to rinse. The slides were then placed in the anti-blue solution, immersed for 3-5s, and rinsed in water. The slides were sequentially immersed in anhydrous ethanol 1, anhydrous ethanol 2, anhydrous ethanol 3, n-butanol 1, n-butanol 2, xylene 1, and xylene 2 for 5 min, and then taken out of the slides, air-dried, and then the tissue was dripped with an appropriate amount of neutral tree resin, covered with coverslips, and left to dry.
